# A minimal RNA substrate with dual fluorescent probes enables rapid kinetics and provides insight into bacterial RNase P active site interactions

**DOI:** 10.1039/d4cb00049h

**Published:** 2024-05-17

**Authors:** Tong Huang, Alexandra Chamberlain, Jiaqiang Zhu, Michael E. Harris

**Affiliations:** a Department of Chemistry, University of Florida Gainesville FL 32608 USA harris@chem.ufl.edu

## Abstract

Bacterial ribonuclease P (RNase P) is a tRNA processing endonuclease that occurs primarily as a ribonucleoprotein with a catalytic RNA subunit (P RNA). As one of the first ribozymes discovered, P RNA is a well-studied model system for understanding RNA catalysis and substrate recognition. Extensive structural and biochemical studies have revealed the structure of RNase P bound to precursor tRNA (ptRNA) and product tRNA. These studies also helped to define active site residues and propose the molecular interactions that are involved in substrate binding and catalysis. However, a detailed quantitative model of the reaction cycle that includes the structures of intermediates and the process of positioning active site metal ions for catalysis is lacking. To further this goal, we used a chemically modified minimal RNA duplex substrate (MD1) to establish a kinetic framework for measuring the functional effects of P RNA active site mutations. Substitution of U69, a critical nucleotide involved in active site Mg^2+^ binding, was found to reduce catalysis >500-fold as expected, but had no measurable effect on ptRNA binding kinetics. In contrast, the same U69 mutations had little effect on catalysis in Ca^2+^ compared to reactions containing native Mg^2+^ ions. CryoEM structures and SHAPE mapping suggested increased flexibility of U69 and adjacent nucleotides in Ca^2+^ compared to Mg^2+^. These results support a model in which slow catalysis in Ca^2+^ is due to inability to engage U69. These studies establish a set of experimental tools to analyze RNase P kinetics and mechanism and can be expanded to gain new insights into the assembly of the active RNase P–ptRNA complex.

## Abstract 2

Bacterial ribonuclease P (RNase P) is a tRNA processing endonuclease that occurs primarily as a ribonucleoprotein with a catalytic RNA subunit (P RNA).

## Graphical abstract

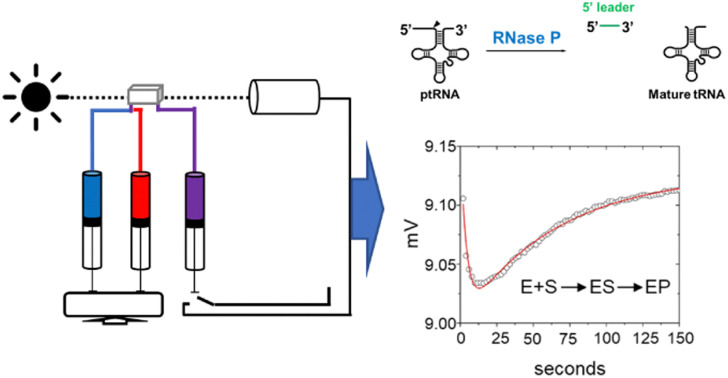

## Introduction

Ribonuclease P (RNase P) is a divalent metal ion dependent endonuclease that processes the 5′ end of tRNAs, SRP RNA, tmRNA, and other small structured RNAs.^1–6^ RNase P occurs throughout phylogeny primarily as a ribonucleoprotein, which in Bacteria is composed of a *ca.* 400 nucleotide catalytic RNA subunit (*rnpB*)^7^ and a smaller protein subunit (*rnpA*, termed C5 in *Escherichia coli*).^8–10^ As one of the first ribozymes discovered, RNase P has served as a model system for exploring RNA catalysis and molecule recognition. The interactions that govern RNase P recognition of precursor tRNA (ptRNA) have been determined by high throughput mutagenesis, structure probing, X-ray crystallography of the *Thermotoga maritima* EP complex, and more recently cryoEM structures of the *E. coli* ES complex, as discussed in several reviews.^8,10–12^ However, a complete model of the reaction cycle is currently lacking, but needed to fully understand how alternative substrates are bound and processed, and to understand the precise role and positioning of divalent ions in catalysis.

**Fig. 1:**
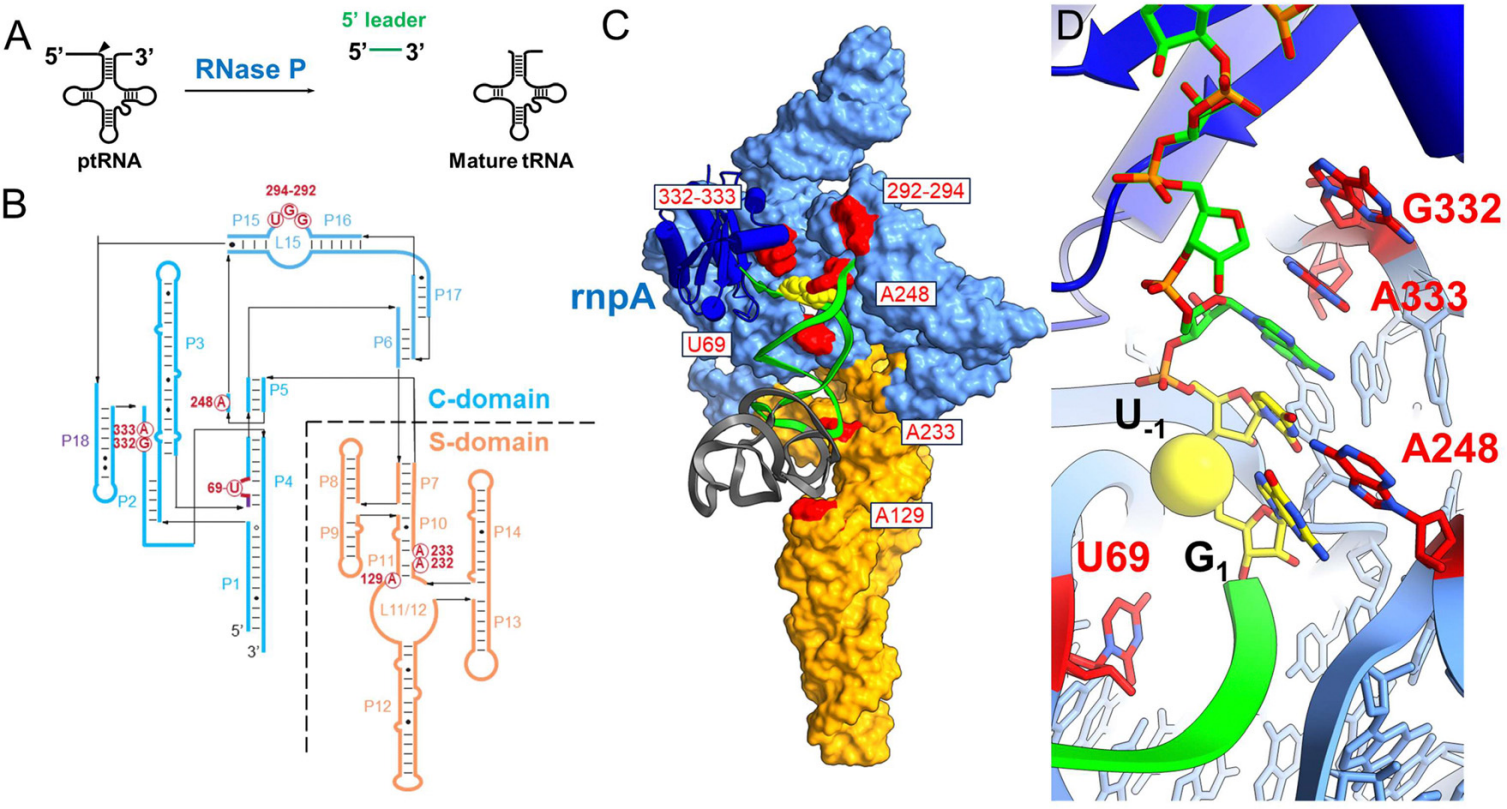
Ribonuclease P structure and interactions with substrate ptRNA. (A) Ribonuclease P (RNase P) catalyzes the maturation of tRNA precursors (ptRNAs) *via* a site-specific endonuclease cleavage to generate the mature tRNA and free 5′ leader product. (C) CryoEM structure of *E. coli* RNase P bound to pre-tRNA^Met^; colored according to panel B. The P RNA subunit consists of the specificity domain (S-domain, orange) and the catalytic domain (C-domain, blue) which includes the P15/16 internal bulge that binds the ptRNA 3′RCCA sequence. (D) Detail of the *E. coli* RNase P holoenzyme active site. The ptRNA, P RNA and C5 are colored the same as in panel C and the reactive phosphoryl group is shown as a yellow sphere. The complementary strand of the acceptor stem including the 3′ ACCA sequence is not shown for clarity.

RNase P recognizes the shape of diverse tRNA substrates by binding the stacked acceptor and T-stems within two domains in the P RNA subunit (Fig. 1).^8,12–14^ The specificity domain (S-domain) binds the T-stem loop *via* A233 and A129 (*E. coli* P RNA numbering) while the 5′-terminal base of the acceptor stem contacts the catalytic domain (C-domain) by stacking on conserved residue A248.^15–17^ A248 can also pair with nucleotide N_−1_ in the substrate 5′ leader (where N_1_ is the first nucleotide of the mature tRNA and cleavage occurs between N_−1_ and N_1_).^18–21^ The 5′ and 3′ ends of ptRNA are splayed and interact with different sites within the C-domain. The 3′ RCC sequence of ptRNA pairs with conserved residues (G292,G293,U294) in the P15/16 internal bulge of RNase P.^22–26^ The nucleotides N_−1_N_−2_ adjacent to the cleavage site connect the acceptor stem and active site *via* a continuous stacking interaction in which they stack upon each other and with A333 in P RNA.^16^ The active site further includes a conserved bulged helix, P4, that positions Mg^2+^ ions that are directly involved in catalysis.^27–31^ Multiple lines of evidence including rapid kinetics and mutagenesis support a two-step binding mechanism involving a conformational change linked to Mg^2+^ binding that is required for catalysis.^19,21,32–34^ Chemical protection and FRET experiments, and cryoEM structures are also consistent with conformational changes in the C-domain that are involved in RNase P function,^16,33,35,36^ but the molecular details are poorly understood. Deeper investigation of the RNase P substrate binding reaction and participation of active site residues is needed to provide the information necessary to develop mechanistic models that relate structure, kinetics, and biological function.

Minimal helical substrates, or minihelix substrates, represent a widely used and valuable experimental tool for investigating RNase P kinetics and active site interactions *in vitro.*^37^ Minihelices have been particularly useful in defining RNase P metal ion cleavage requirements,^24,34,38,39^ specificity,^40–44^ for systematic investigation of functional groups at the cleavage site G_1_–C_72_ and N_−1_ positions,^21,45–48^ small molecule screening,^49^ and RNase P engineering.^50,51^ Similarly, small bipartite model substrates formed from annealing short oligonucleotides are also efficient substrates for bacterial RNase P.^43,52,53^ However, most prior studies using model substrates primarily investigated reactions with the RNA subunit alone at high monovalent and divalent ion concentrations, which makes quantitative comparison and relating results to the biological context more challenging. Advantageously, synthetic RNAs are readily modified with fluorescent labels, nucleotide analogs, and isotopic substitutions and applied to RNase P holoenzyme reactions to provide deeper mechanistic insights into the kinetics of substrate binding, conformational changes, and transition state stabilization.

Here, we describe a minimal duplex substrate (MD1) for bacterial RNase P that facilitates incorporation of fluorescent probes permitting its use for mechanistic studies of substrate binding and catalysis. We used this tool to quantify the effects of mutations at the key active site residues in P RNA and provide evidence that G332 and A333, previously implicated in sequence specific interactions, contribute little to binding kinetics for this substrate. Remarkably, we found that mutations at U69 in P4 that were previously shown to result in large defects on RNase P activity, had minimal impact on substrate binding kinetics. Surprisingly, the effects of U69 mutation on catalysis were suppressed in Ca^2+^ compared to Mg^2+^, which correlates with greater flexibility of U69 in Ca^2+^ based on cryoEM structural analysis and SHAPE probing. Importantly, the results suggest that slow catalysis in Ca^2+^ is due to an inability to engage in productive binding interactions with U69 and not intrinsic differences in transition state stabilization relative to Mg^2+^.

## Results

### Single turnover reaction kinetics of MD1 processing by *E. coli* RNase P

**Fig. 2:**
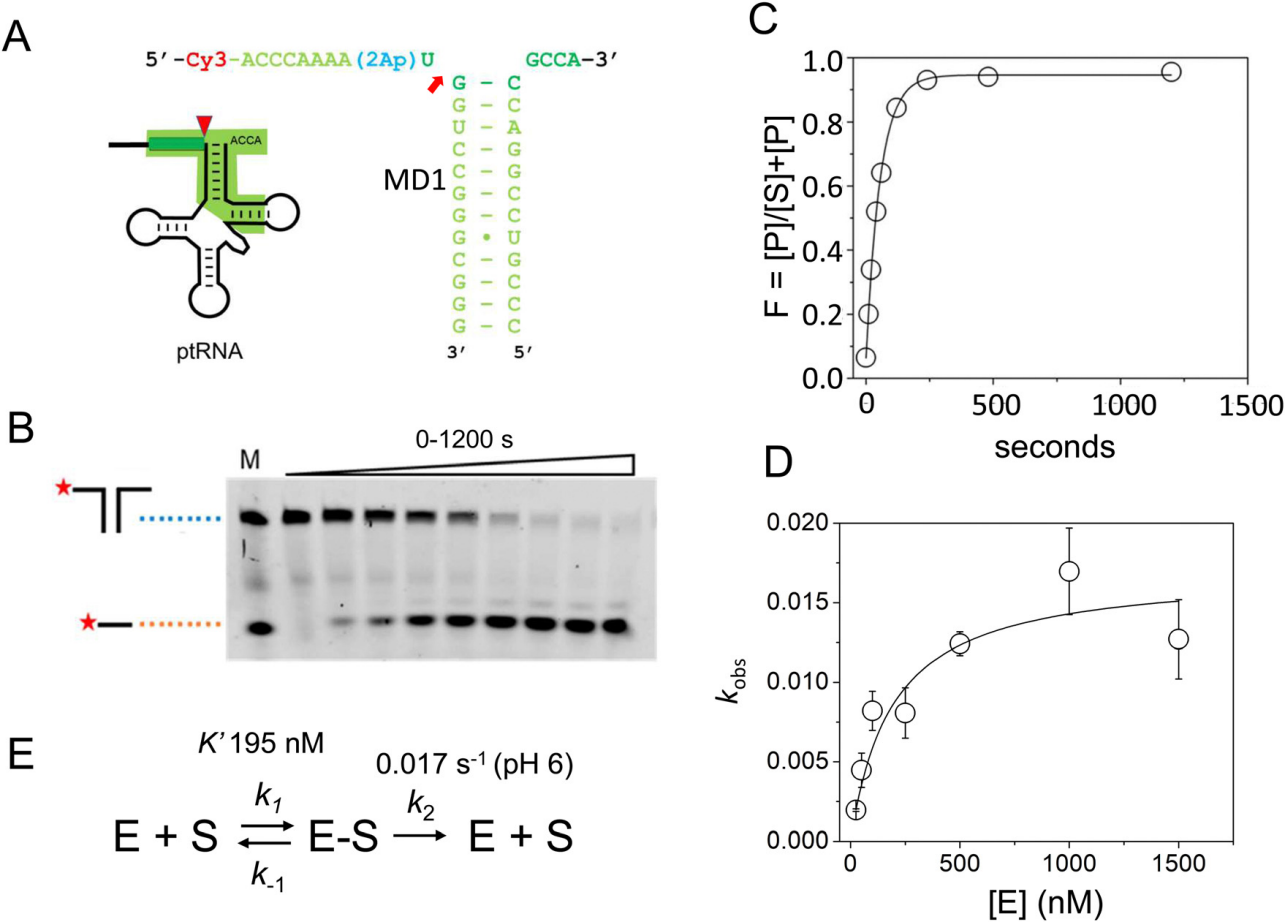
Sequence and single turnover kinetics of MD1 cleavage by *E. coli* RNase P. (A) Design and sequence of MD1 substrate consisting of two annealed synthetic RNA oligonucleotides. The nucleotides in ptRNA consisting of the stacked acceptor and TΨC stem included in MD1 are boxed, and a red arrow indicates the RNase P cleavage site. (B) PAGE analysis of products from a reaction containing 50 nM 5′Cy3-MD1 and 1 μM *E. coli* RNase P. The positions in the gel of the annealed MD1 duplex and the 5′ fragment generated by RNase P cleavage are shown on the left. Lane M contains equal amounts of the annealed MD1 substrate and a synthetic RNA oligo matching the expected cleavage product (Table 1). (C) Plot of fraction (F) of product formed from total substrate concentration ([P]/([P] + [S])) determined by phosphorimager analysis *versus* time. These data are fit to an exponential function (eqn (1), Methods) to determine *k*_obs_. (D) Dependence of *k*_obs_ for MD1 single turnover reactions as a function of increasing *E. coli* RNase P concentration. These data are fit to an equation for Scheme 1 involving equilibrium binding followed by the substrate cleavage step (eqn (2), Methods; *k*_c_ = 0.017 s^−1^; *K*′ = 189 nM) as shown in panel E and described in the text.

The acceptor and T-stems of ptRNA are contacted by RNase P while the remainder of tRNA is dispensable for binding. However, impaired coaxial stacking of the acceptor and T-stems, or omission of the T-loop of ptRNA reduces cleavage rates *e.g*.^37,44,54^*In vitro* studies show that, in general, truncated substrates bind with lower affinity due to loss of important enzyme–substrate contacts, although there are intriguing differences in the ability of RNase P enzymes to accept minimal substrates that are not fully understood.^44^ The MD1 substrate consists of a 21 nucleotide RNA oligonucleotide (5′2Ap(-2)-MD1) that forms a 12 basepair continuous helix with a complementary 16 nucleotide RNA (3′MD1) (Table 1, Fig. 2). When annealed, these two oligos form the stacked acceptor and T-stems of a well characterized *B. subtillis* tRNA^Asp^ substrate including nine nucleotides of genomically encoded 5′ leader and 3′ GCCA sequence.^55^ Two different modifications were used to incorporate fluorescent probes for analysis of MD1 binding and kinetics. The 5′Cy3Ap(-2)-MD1 RNA contains a 5′ Cy3 modification at the end of the 5′ leader and allows MD1 and its 5′ leader cleavage product to be visualized by denaturing PAGE, and binding kinetics to be measured using fluorescence anisotropy. 2-Aminopurine (Ap) is an intrinsically fluorescent nucleobase that is quenched by base stacking.^56^ Thus, since N_−2_ forms a stacking interaction with A333 in the *E. coli* RNase P active site,^16^ we reasoned that a 2Ap modification at this position should be sensitive to such active site conformational changes. Accordingly, 2Ap was included at N_−2_ to monitor changes in the environment of this nucleotide position.

**Table 1:** Mini-duplex (MD1) oligonucleotides

ssRNA	Sequence	Length (nt)
3′MD1	5′ C.C.C.G.U.C.C.G.G.A.C.C.G.C.C.A 3′	16
5′ Ap(-2)-MD1	5′ Cy3.A.C.C.C.A.A.A.2Ap.U.G.G.U.C.C.G.G.G.C.G.G.G 3′	21
5′ Cy3-MD1	5′ A.C.C.C.A.A.A.2Ap.U.G.G.U.C.C.G.G.G.C.G.G.G 3′	21
5′Cy3Ap(-2)-MD1	5′ Cy3.A.C.C.C.A.A.A.2Ap.U.G.G.U.C.C.G.G.G.C.G.G.G 3′	21
5′Cy3Ap(-2)BHQ-MD1	5′ Cy3.A.C.C.C.A.A.A.2Ap.U.G.G.U.C.C.G.G.G.C.G.G.G.BHQ2 3′	21
MD.L	5′ Cy3.A.C.C.C.A.A.A.A.U	9

First, we determined the single turnover reaction kinetics and specificity for MD1 cleavage by *E. coli* RNase P under reaction conditions of 0.1 M NaCl, 17.5 mM Mg^2+^, and MES pH 6. These monovalent and divalent ion concentrations are optimal for the multiple turnover reaction (*k*_cat_/*K*_m_) of native *E. coli* RNase P with an *E. coli* ptRNA^Met^ substrate.^57^ Non-optimal conditions of low pH were used to slow the catalysis so that the cleavage step could be measured by manual pipetting.^58^ The kinetics of formation of the 5′ leader product were analyzed by denaturing PAGE and phosphorimager analysis. The 5′ cleavage product co-migrated with a synthetic 5′-Cy3 labeled RNA oligonucleotide of the same sequence (MD.L), which is consistent with hydrolysis at the correct phosphodiester bond between U(-1) and G(1) (Fig. 2B). The kinetic data fit well to a single exponential function (eqn (1), Methods) over a range of 50–1500 nM RNase P (Fig. 2C).

**Scheme 1:**
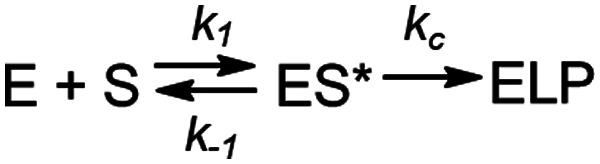


The observed pseudo-first-order rate constant (*k*_obs_) increased with RNase P concentration until a plateau is reached at 500 nM (Fig. 2D). The data are consistent with single substrate enzyme mechanism involving a second-order reaction (substrate binding) followed by irreversible product formation (Scheme 1).

The dependence of *k*_obs_ on RNase P concentration was fit to this mechanism (eqn (2), Methods) in order to estimate the magnitude of *k*_c_ and *K*′ (where *K*′ = *k*_−1_/*k*_1_) (Fig. 2E). The rate constant *k*_c_ for conversion of ES to form EP was observed to be *ca.* 0.017 s^−1^, which is consistent with the value of 0.02 s^−1^ previously reported by Kirsebom and colleagues for a similar pATSerUG minihelix substrate,^59^ and 0.0143 s^−1^ measured for a 3 bp minihelix substrate under similar conditions.^48^ These values for *k*_c_ are also consistent with the value of 0.028 s^−1^ measured for native RNase P processing of an optimal ptRNA^Met^ substrate.^60^ A *K*′ of 189 nM was obtained by fitting the data in Fig. 2D to eqn (2) (Methods). This value is similar to the *K*_d_,_app_ for binding of pATSerUG stem-loop substrate of 92 nM and a smaller substrate pMini3bpUG that has a *K*_d_,_app_ of 150 nM, both measured in 40 mM Ca^2+^.^34^ Thus, MD1 is efficiently processed by *E. coli* RNase P with monophasic single turnover kinetics with reaction rate and apparent affinity matching comparable minimal substrates measured under similar reactions conditions.

### Analysis of MD1 binding and cleavage using stopped flow fluorescence

Next, we used stopped-flow fluorescence to investigate the kinetics of binding and catalysis, and to measure the individual rate constants for association and dissociation in Scheme 1. The recent cryoEM structure of the *E. coli* RNase P complex with ptRNA showed that the first two nucleotides in the 5′ leader sequence, N_−2_N_−1_, are bound in a pocket between conserved active site nucleotides A248 and A333.^16^ We reasoned that positioning 2Ap near the substrate cleavage site would be sensitive to binding and cleavage by RNase P. Mutagenesis experiments showed that A is the optimal nucleobase N_−2_ and G had only a small effect on *k*_cat_/*K*_m_.^16,61^ These results suggest that 2Ap at N_−2_ in the context of the 5′Ap(-2)-MD1 substrate will fit similarly into the same site in RNase P and report on association and cleavage kinetics.

**Fig. 3:**
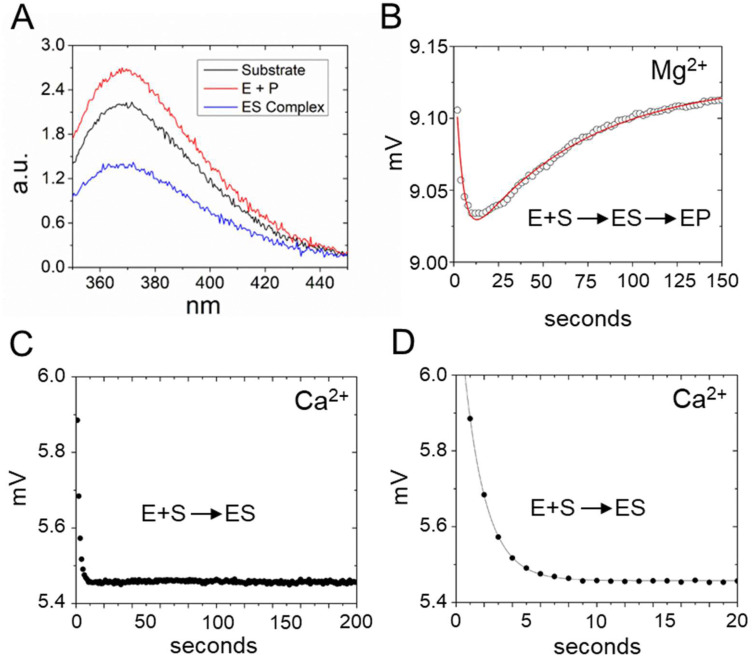
Analyses of 5′2Ap(-2)-MD1 reaction with *E. coli* RNase P using equilibrium and stopped-flow fluorescence spectroscopy. (A) Equilibrium fluorescence spectra (*λ*_ex_ = 300 nm) of 100 nM 5′2Ap(-2)-MD1 (substrate, black line), and 100 nM 5′2Ap(-2)-MD1 combined with 1 μM *E. coli* RNase P in 0.1 M NaCl, 17.5 mM MgCl_2_, 0.1 mM EDTA (product, red line), and 100 nM 5′2Ap(-2)-MD1 combined with 1 μM *E. coli* RNase P, in the same reaction buffer with 17.5 mM CaCl_2_ instead of MgCl_2_ (ES complex, blue line). (B) Stopped flow fluorescence experiment measuring total fluorescence using a 320 nm high pass filter to measure total fluorescence above the cut off wavelength and excitation at 300 nm using an LED source as described in Methods. Time-dependent change in fluorescence in detector voltage upon mixing to achieve final concentrations of 100 nM 5′2Ap(-2)-MD1 and 1 μM *E. coli* RNase P. The data fit a double exponential function (eqn (3), red line) as described in Methods (0.16 s^−1^ for *k*_obs1_ and 0.024 s^−1^ for *k*_obs2_). (C) Time-dependent change in fluorescence upon mixing to achieve 100 nM 5′2Ap(-2)-MD1 and 1 μM *E. coli* RNase P in the same reaction buffer with 17.5 mM CaCl_2_ instead of MgCl_2_. These data were fit to a single exponential function (eqn (1), *k*_obs_ = 0.64 s^−1^). (D) Shorter time scale for the same reaction in panel C showing the initial decrease in fluorescence signal.

To test the effect of RNase P binding on 5′Ap(-2)-MD1 fluorescence, we first measured the steady-state emission spectra of 100 nM substrate in the absence and presence of 1 μM RNase P (Fig. 3A). Ca^2+^ was used as the divalent ion, which slows catalysis by *ca.* 500-fold, allowing the emission spectrum of the ES complex to be acquired.^62^ The emission spectrum of 5′Ap(-2)-MD1 (*λ*_ex_ = 310 nm) is characteristic of 2-aminopurine with a broad peak with a maximum at *ca.* 365 nm.^56,63^ We observed that fluorescence intensity decreased upon addition of RNase P to a final concentration of 1 μM in the presence of 17.5 mM Ca^2+^, while the signal increased to even higher levels than for MD1 alone after 1 h of incubation at 17.5 mM Mg^2+^ to completely convert substrate to product. These results are consistent with quenching of the 2Ap at N_−2_ in the ES complex and recovery of the signal upon substrate cleavage to form EP or rapid dissociation of the 5′ leader.

To measure the kinetics of the 5′Ap(-2)-MD1 fluorescence during the reaction we used stopped-flow spectroscopy (Fig. 3B). RNase P and MD1 solutions were mixed to achieve final concentrations of 500 nM and 100 nM, respectively. Under these conditions, a rapid decrease in fluorescence was observed initially, followed by a slower increase in the fluorescence signal. We reasoned that the initial rapid decrease in signal likely reflects binding to form ES, which is consistent with the steady-state fluorescence results. The slower increase in fluorescence occured over a time scale similar to product formation observed by PAGE. To estimate the rate constants for the first and second phases (*k*_obs1_ and *k*_obs2_, respectively) these data were fit to a double exponential function (eqn (3)). Values of 0.16 s^−1^ for *k*_obs1_ and 0.024 s^−1^ for *k*_obs2_ were measured for the data in Fig. 3B. To test whether the two fluorescence transients represent binding and catalysis, we used Ca^2+^ as the divalent ion which slows catalysis with minimal effect on binding steps.^62^ When Ca^2+^ was used as the divalent ion, a fast decrease in 2Ap fluorescence was still observed (Fig. 3C and D). However, the subsequent increase in signal observed with Mg^2+^ was absent for up to 200 seconds. Under these conditions, there was minimal product formation detectable by PAGE (see below). Thus, the rapid decrease in fluorescence in Fig. 3B is likely to reflect binding or a conformational change after binding, while the slower increase in fluorescence is attributable to substrate cleavage.

**Fig. 4:**
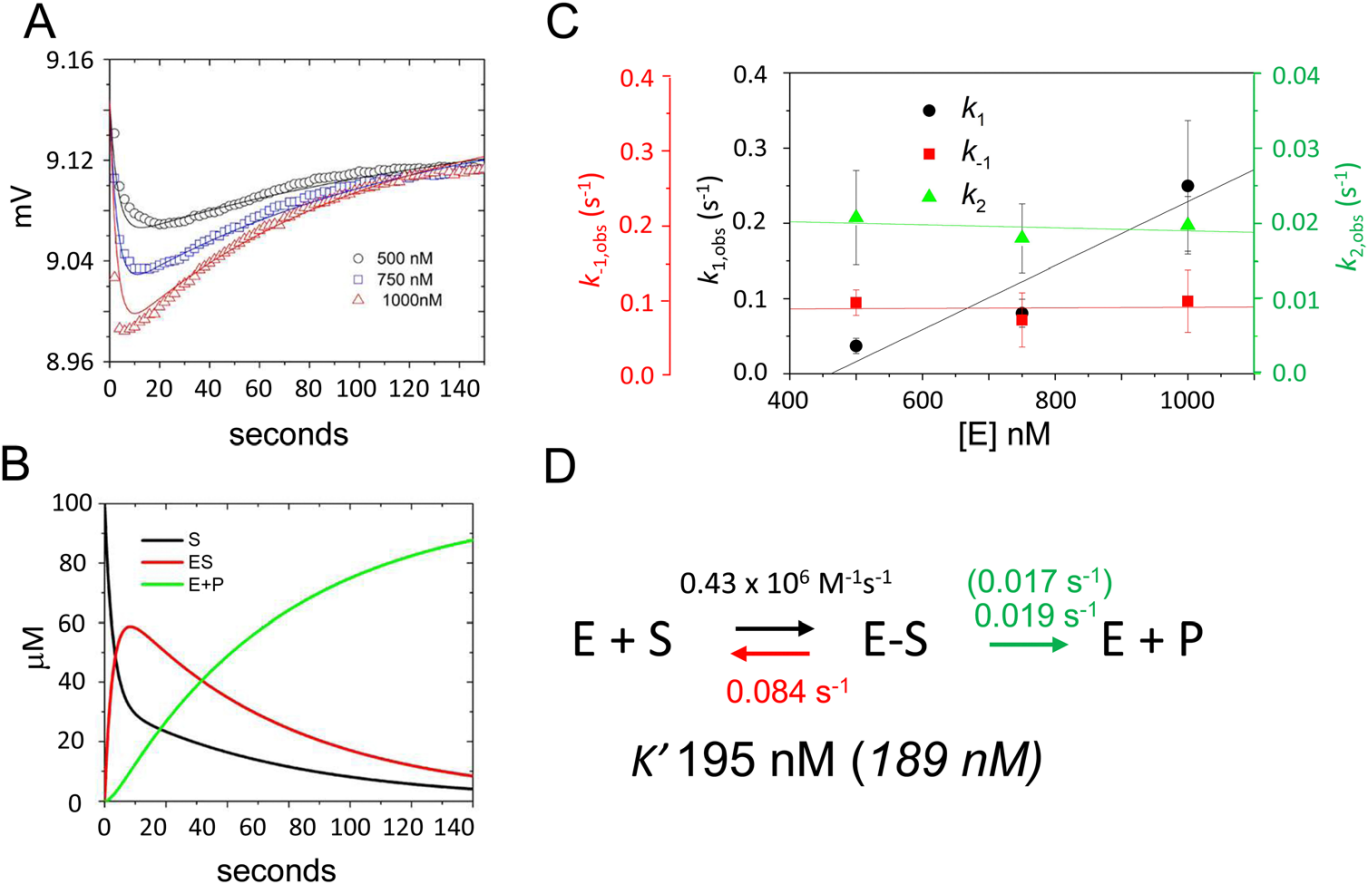
Global fitting determination of *k*_1_, *k*_−1_, and *k*_2_ for 5′Ap(-2)-MD1 cleavage by RNase P. (A) Time-dependent change in fluorescence upon mixing substrate and enzyme to achieve final concentrations of 100 nM 5′2Ap(-2)-MD1 and 0.5 μM (black line and data), 0.75 μM (blue line and data), and 1 μM *E. coli* RNase P (red line and data). The data fit the rate equation for Scheme 1 as described in Methods. (B) Kinetics of the individual changes in S (black), ES (red), and EP (green) for fitting Scheme 1 to the 100 nM 5′2Ap(-2)-MD1 and 0.75 μM *E. coli* RNase P data. (C) Observed dependence of average values for *k*_1_ (black circles), *k*_−1_ (red squares), and *k*_2_ (green triangles) for reactions containing 0.5 μM, 0.75 μM, and 1 μM *E. coli* RNase P. The data are fit to a linear function in order to illustrate reaction order. (D) Summary of *k*_1_, *k*_−1_, *k*_2_ and *K*′ values obtained from global fitting to the kinetic model shown in Scheme 1; values in parentheses were inferred from single turnover kinetics using PAGE analysis.

To validate this model and estimate rate constants for individual reaction steps we acquired time-dependent fluorescence data using *E. coli* RNase P concentrations of 500 nM, 750 nM, and 1000 nM (Fig. 4A). The magnitudes of *k*_1_ and *k*_−1_ could be evaluated only under a limited range of RNase P concentrations since the decrease and subsequent increase in signal due to binding and catalysis largely offset each other below 500 nM. At RNase P concentrations greater than 1000 nM binding becomes difficult to measure accurately due to instrument dead time limitations (*i.e.* the interval between mixing and the start of data collection). Nonetheless, these data were sufficient to estimate reaction rate constants by global fitting to the kinetic model shown in Scheme 1, assuming scaling factors to account for the expected signal differences for S, ES, and EP (Fig. 4A). A plot of *k*_1_, *k*_−1_, and *k*_2_ values obtained from global fitting *versus* RNase P concentration shows that *k*_2_ and *k*_−1_ were essentially independent of RNase P concentration consistent with unimolecular reactions. In contrast, the magnitude of *k*_1_ increased with increasing enzyme concentration, which is characteristic of bimolecular association (Fig. 4C). The data are consistent with a second-order rate constant for substrate association, *k*_1_, of 0.43 × 10^6^ M^−1^ s^−1^. The fitted value for the cleavage step, *k*_2_, of 0.019 s^−1^ matches the value of 0.017 s^−1^ measured by single turnover kinetics using PAGE (Table 2). The value of *k*_−1_ of 0.084 s^−1^ is consistent with a near rapid equilibrium single turnover mechanism in which the *K*′ of 184 nM observed by fitting the enzyme concentration dependence of *k*_obs_ (Fig. 2E) is equivalent to *K*_d_ = *k*_−1_/*k*_1_. Assuming this mechanism, we note that the observed rate constant for association *k*_1_ of 0.43 × 10^6^ M^−1^ s^−1^ and dissociation rate constant *k*_−1_ of 0.084 s^−1^ from global fitting predicts a *K*′ of 195 nM that is within error of 189 nM measured by single turnover kinetics using PAGE.

**Table 2:** Single turnover kinetic parameters *K*′ and *k*_c_ measured for WT and mutant *E. coli* RNase P enzymes

RNase P	*K*′ (nM)	*k*_c_ (s^−1^) Mg^2+^	*k*_c_ (s^−1^) Ca^2+^
WT	190	0.017(3)	0.000024(2)
G332U	141	0.013(6)	—
A333U	108	0.011(1)	—
U69A	143	0.0013(1)	0.000016(4)
C70U	435	0.0005(1)	0.00003(1)

**Fig. 5:**
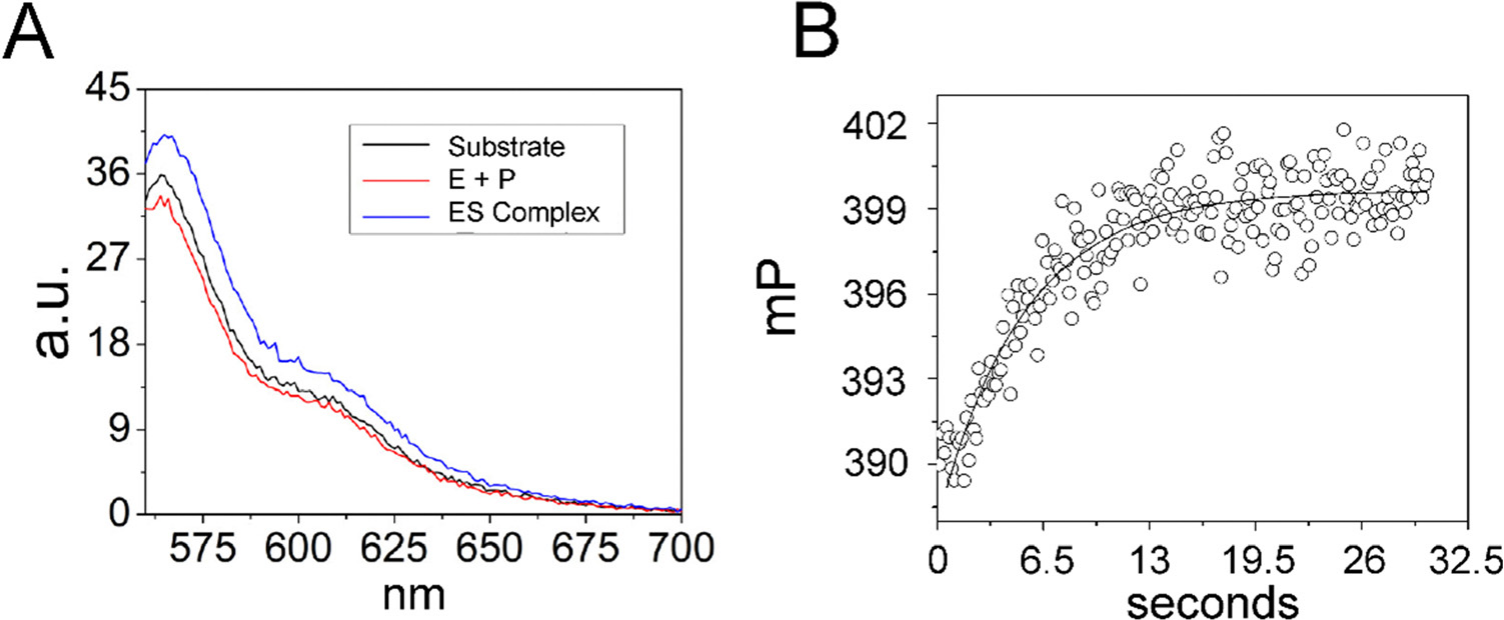
Analyses of 5′Cy3Ap(-2)-MD1 reaction with *E. coli* RNase P using equilibrium fluorescence spectroscopy and stopped-flow fluorescence anisotropy. (A) Steady state fluorescence spectra (*λ*_ex_ = 550 nm) of 100 nM 5′Cy3Ap(-2)-MD1 (substrate, black line), and 100 nM 5′Cy3Ap(-2)-MD1 combined with 1 μM *E. coli* RNase P in 17.5 mM MgCl_2_, (product, red line), and 100 nM 5′Cy3Ap(-2)-MD1 combined with 1 μM *E. coli* RNase P, in 17.5 mM CaCl_2_ instead of MgCl_2_ (ES complex, blue line). (B) Stopped flow fluorescence anisotropy experiment measured using 570 nm high pass filters and excitation at 535 nm using an LED source as described in Methods. Time-dependent change in fluorescence polarization (mP, millipolarization) upon mixing to achieve final concentrations of 100 nM 5′Cy3Ap(-2)-MD1 and 1 μM *E. coli* RNase P. The data are fit to a single exponential function (eqn (1), Methods; *k*_obs_ = 0.23 s^−1^).

Since analysis of 5′Ap(-2)-MD1 kinetics using 2Ap fluorescence was limited to a narrow concentration range we took advantage of the 5′-Cy3 modification of 5′Cy3Ap(-2)-MD1 to further test whether the initial fluorescent transient detected by 2Ap fluorescence accurately measures substrate association kinetics. We reasoned that due to the small molecular weight of MD1 as compared to the much larger RNase P, association kinetics for MD1 should be detectable by fluorescence anisotropy. Therefore, we collected the steady-state emission spectra of 5′Cy3Ap(-2)-MD1 free in solution, bound to RNase P in the presence of Ca^2+^, and under rapid cleavage conditions in Mg^2+^. In contrast to the significant changes in the 2Ap signal, the Cy3 fluorescence intensity was minimally sensitive to binding and cleavage by RNase P (Fig. 5A). However, when 5′Cy3Ap(-2)-MD1 was combined with RNase P to achieve final concentrations of 100 nM substrate and 1000 nM enzyme, a rapid increase in anisotropy was observed that is consistent with the association of MD1 with the significantly larger ribonucleoprotein enzyme. The increase in anisotropy occurred with an observed pseudo-first-order rate constant of *ca.* 0.23 s^−1^, similar to the observed *k*_1_ obtained with the 5′Ap(-2)-MD1 substrate at the same concentration (1000 nM) (Fig. 5B).

The rate constants for association and dissociation estimated by global fitting of stopped-flow fluorescence data using 5′Cy3Ap(-2)-MD1 reflect a small commitment to catalysis (*C*_f_ = *k*_2_/*k*_−1_ = 0.017 s^−1^/0.084 s^−1^ = 0.2).^64^ Thus, the *K*′ value determined from fitting single turnover data approximates the dissociation constant for binding (*K*_d_). Using this framework, we next investigated the effects of a series of active site mutations to quantify their contributions to binding and catalysis, which will help to guide a detailed inhibitor characterization and structure-function studies at a chemical level.

### Multiple turnover reaction kinetics of MD1 processing by *E. coli* RNase P

**Fig. 6:**
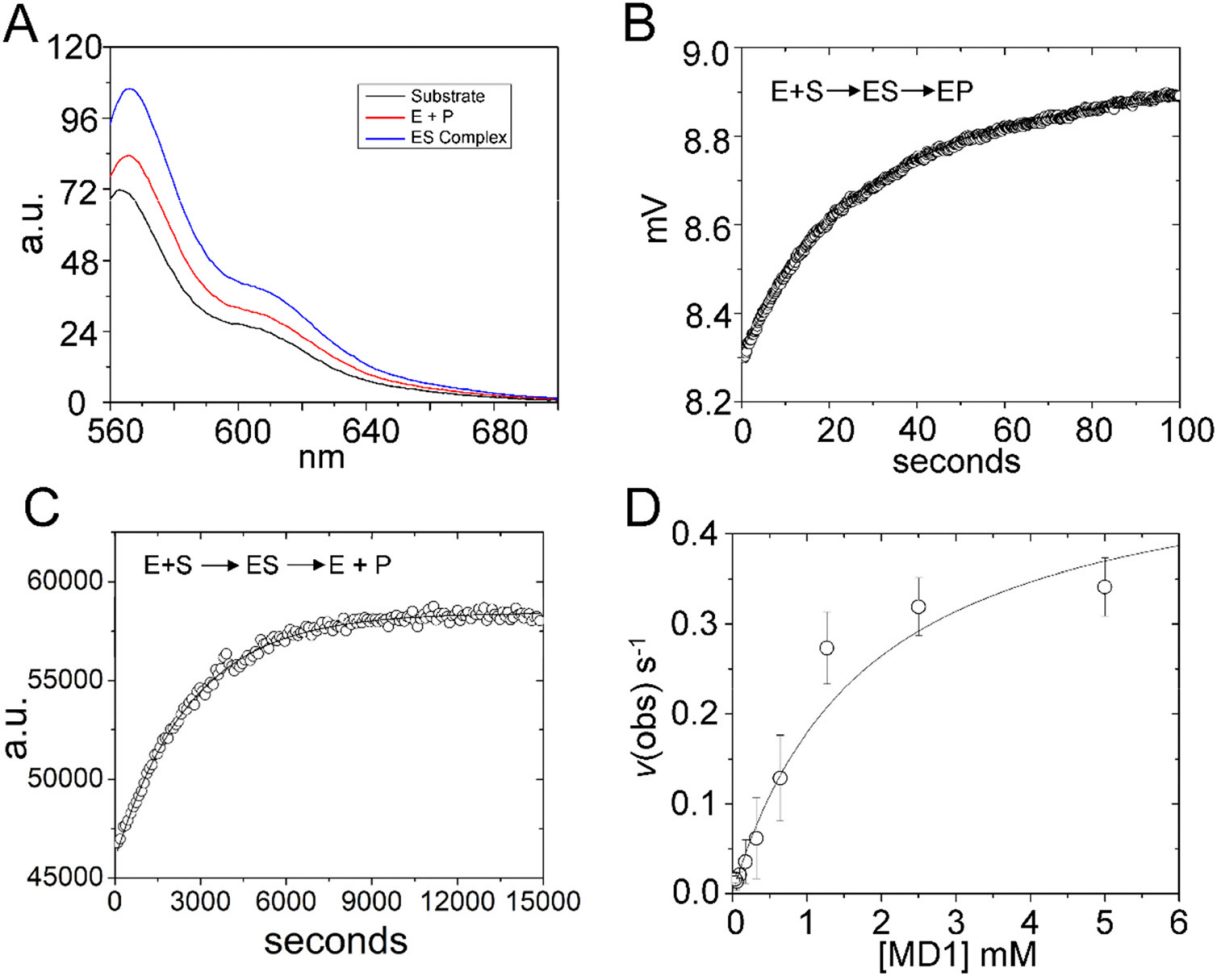
Multiple turnover kinetics of 5′Cy3Ap(-2)BHQ-MD1 cleavage by *E. coli* RNase P. (A) Steady state fluorescence spectra (*λ*_ex_ = 550 nm) of 100 nM 5′Cy3Ap(-2)BHQ-MD1 (substrate, black line), and 100 nM 5′Cy3Ap(-2)BHQ-MD1 combined with 1 μM *E. coli* RNase P in 17.5 mM MgCl_2_, (product, red line), or with 1 μM *E. coli* RNase P in 17.5 mM CaCl_2_ (ES complex, blue line). (B) Stopped flow fluorescence experiment measuring total fluorescence using a 570 nm high pass filter and excitation at 535 nm using an LED source as described in Methods. Time-dependent change in fluorescence in detector voltage upon mixing to achieve single turnover conditions with final concentrations of 100 nM 5′Cy3Ap(-2)BHQ-MD1 and 1 μM *E. coli* RNase P. The data were fit to a single exponential function (eqn (1)) where *k*_obs_ = 0.026 s^−1^. (C) Multiple turnover kinetics of Cy3 fluorescence increase upon cleavage measured using plate reader as described in Methods. Time-dependent change in fluorescence intensity upon mixing to achieve final concentrations of 50 nM–5 μM 5′Cy3Ap(-2)BHQ-MD1 and 10 nM *E. coli* RNase P. (D) Kinetics data are fit to saturation binding equation consistent with Scheme 1 to estimate *k*_cat_ (0.5 s^−1^) and *K*_m_ (1.8 μM) (eqn (2), Methods).

Steady state kinetics are important for understanding biological specificity, competition between alternative substrates, and for screening assays. Therefore, to determine the Michaelis–Menten kinetic parameters *k*_cat_, *K*_m_ and *k*_cat_/*K*_m_ for MD1 cleavage, we positioned a BHQ2 modification at the 3′ end of 5′Cy3Ap(-2)-MD1 to quench the fluorescence of Cy3 in the substrate and undergo an increase in fluorescence upon formation of the cleaved product. Accordingly, we collected the steady-state emission spectra of 5′Cy3Ap(-2)BHQ-MD1 free in solution under reaction conditions, bound to RNase P in the presence of Ca^2+^ to slow catalysis, and under cleavage conditions in Mg^2+^ (Fig. 6A). While there is a small change in fluorescence intensity upon formation of ES, a much larger increase in fluorescent signal occurs concomitant with formation of EP.

Using this information, we confirmed that the increase in Cy3 signal upon product formation observed with 5′Cy3Ap(-2)BHQ-MD1 is consistent with the kinetics of this step observed by monitoring the 2Ap modification at N_−2_. Stopped flow fluorescence experiments measuring Cy3 emission upon mixing 5′Cy3Ap(-2)BHQ-MD1 and RNase P to achieve final concentrations of 100 nM and 1000 nM, respectively, showed the expected large increase in fluorescent signal that is associated with product formation. The increase in signal occurred with an observed rate constant of 0.026 ± 0.05 s^−1^ (Fig. 6B), consistent with the *k*_2_ of 0.019 s^−1^ from global fitting of 5′Ap(-2)-MD1 fluorescence (Fig. 4). In order to determine the multiple turnover kinetics of MD1 we took advantage of the large signal-on change in Cy3 fluorescence of 5′Cy3Ap(-2)BHQ-MD1 upon cleavage by adapting it to a plate reader assay. As described in Methods, individual wells contained aliquots of substrate at different concentrations that were mixed with enzyme using an integrated injector. Individual time courses of the increase in fluorescence due to product formation were recorded for reactions containing constant (50 nM) 5′Cy3Ap(-2)BHQ-MD1 and increasing concentrations of 5′Ap(-2)-MD1. The primary data for a reaction containing 1 μM substrate is shown in Fig. 6C. The initial rates determined from individual time courses were fit to the Michaelis–Menten equation to estimate *k*_cat_ and *K*_m_ (Fig. 6D). The value of 0.5 ± 0.1 s^−1^ for *k*_cat_ at pH 8 is 20–25 fold faster than the value of *k*_c_ of 0.017–0.022 s^−1^ measured at pH 6.0. This difference is consistent with the pH dependence of RNase P cleavage which follows general base catalysis with a kinetic p*K*_a_ at pH 6.5–8.^32,65^ The value of 1.8 ± 0.4 μM for *K*_m_ is also significantly higher than the magnitude of the analogous quantity *K*′ determined using single turnover kinetics. Importantly, the *K*′ measured at pH 6 was shown to approximate *K*_d_ = *k*_−1_/*k*_1_, while *K*_m_ = (*k*_−1_ + *k*_cat_)/*k*_1_. Therefore, the large magnitude of *k*_cat_ contributes to the comparatively large *K*_m_ compared to *K*′, which more closely reflects equilibrium binding affinity.

### Differential effects of mutations at active site nucleotides on binding and catalysis

**Fig. 7:**
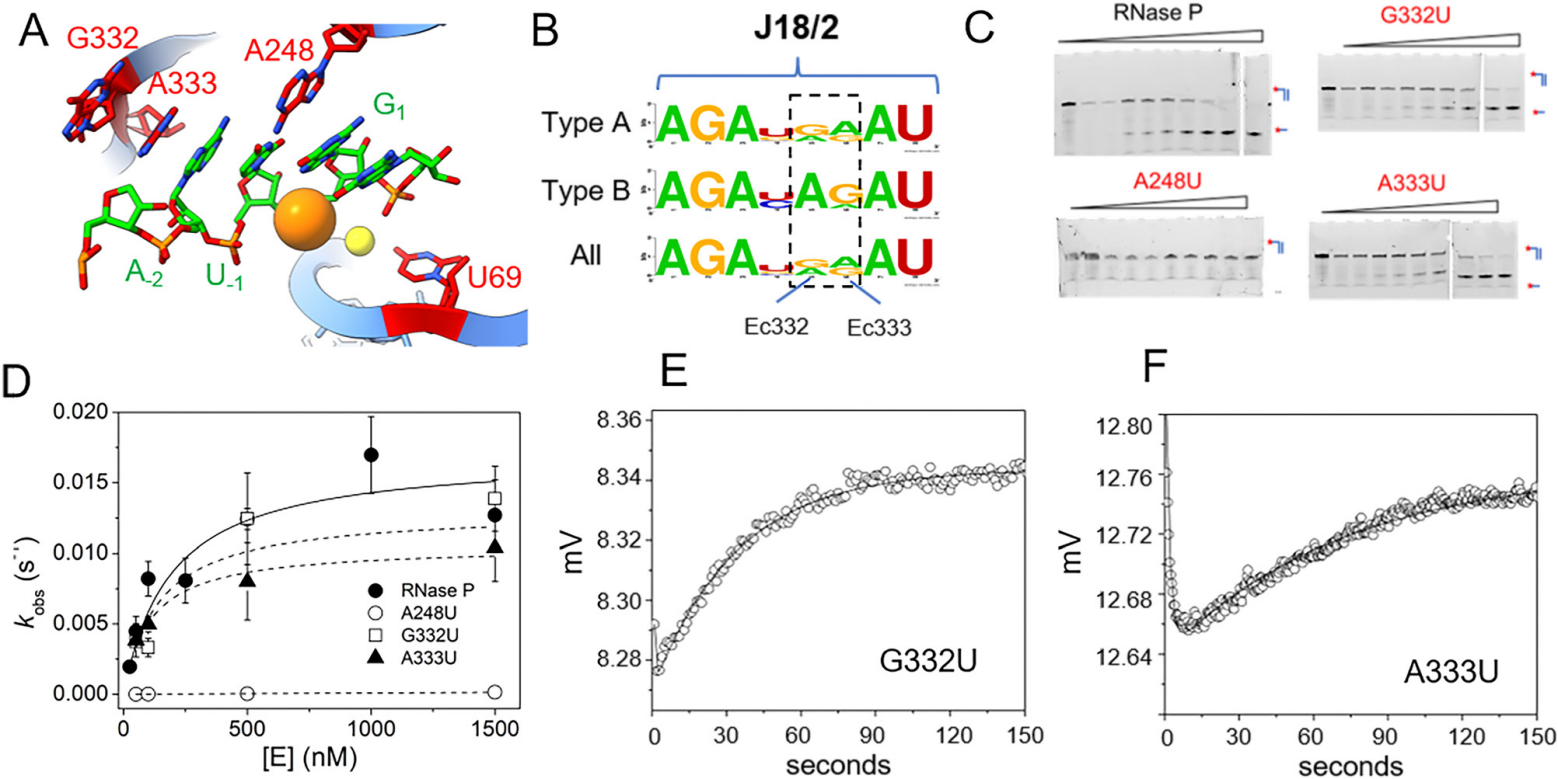
Effects of A248U, G332U, and A333U mutation on *E. coli* RNase P binding and cleavage kinetics. (A) RNase P active site indicating the positions of the cleavage site between U_−1_ and G_1_ (green), and the active site residues A248, G332, and A333 in P RNA (red), the C5 protein subunit is omitted for clarity. (B) Sequence conservation logo of J18/2 from Type A, Type B, and all seed alignment *rnpB* gene sequences in the Rfam database. The positions homologous to nucleotides G332 and A333 in *E. coli* RNase P are highlighted by a dashed box. (C) Denaturing PAGE analysis of the cleavage products of 5′Cy3-MD1 for reactions with RNase P and mutants A238U, G332U, and A333U. (D) Plot of *k*_obs_ for the single turnover reactions of 5′Cy3-MD1 with A248U (open circles), G332U (open squares), and A333U (filled triangles). The data fit an equation for the dependence of *k*_obs_ on [E] according to Scheme 1 as described in Methods (Table 2). (E) and (F) stopped flow fluorescence experiment measuring total fluorescence using a 320 nm high pass filter and excitation at 300 nm with an LED source as described in Methods. Time-dependent change in fluorescence in detector voltage upon mixing to achieve final concentrations of 100 nM 5′2Ap(-2)-MD1 and 1 μM G332U (E) or A333U (F) mutant RNase P. The data were fit to eqn (3) to estimate *k*_obs2_ (*i.e. k*_c_) (G332U *k*_obs2_ = 0.023 s^−1^, A333U *k*_obs2_ = 0.013 s^−1^).

Using MD1 as a substrate we measured the effects of mutations in *E. coli* P RNA at nucleotides contacting the ptRNA cleavage site in the ES complex or involved in catalytic metal ion binding. As illustrated in Fig. 1 the 3′RCCA sequence common to tRNA binds to the P15/16 internal loop between P15 and P16, and distal nucleotides in the 5′ leader are contacted by rnpA protein. With respect to nucleotides directly flanking the cleavage site, three separate regions of P RNA combine to form the active site. These include A248 in J5/15, G332 and A333 in J18/2, and a divalent metal binding site in P4 involving G68 and U69 (Fig. 7A). The interactions between A248 and N_−1_ are understood in some detail.^20,21,66^ A recent cryoEM structure of *E. coli* RNase P bound to ptRNA shows an optimal U_−1_, which is the optimal base at this position, contacts the Hoogstein edge of A248 with a distance of 2.8 Å between the U_−1_ O4 and N6 of A248.^16^ The roles of G332 and A333 are less well understood, but likely contribute to binding, recognition, and/or positioning of the ptRNA leader. The *E. coli* RNase P–ptRNA complex structure reveals that the N_−1_ and N_−2_ bases stack together in a pocket formed by A248, A333, and P4. The leader nucleotides N_−3_ and N_−4_, are in position to contact the Hoogstein edges of G332 and A333, although the precise interactions are not resolved.^16^ The cryoEM density of the ES complex indicates flexibility distal to N_−2_ of ptRNA, and the structural details and functional importance of interactions further out in the 5′ leader remain unclear. Therefore, we used the MD1 substrate to compare the effects of mutation at A248, G332, and A333 on binding affinity, kinetics, and catalysis.

Previous studies of *E. coli* RNase P demonstrated that A248G and A248C mutations had relatively minimal effects on binding and catalysis in reactions using high monovalent ion concentration where the P RNA alone is catalytic.^20,66^ In contrast, a A248U mutation exhibited only a 2–6-fold effect on catalysis in previous studies, but ranges from a 300-fold impact on binding at 25 mM Mg^2+^ to a smaller effect at higher concentrations (800 mM Mg^2+^).^20,66^ Similarly, mutations in J18/2 have also been analyzed for P RNA alone reactions at high ionic strength conditions,^67^ but their contribution to RNase P holoenzyme reactions is poorly understood. The positions homologous to G332 and A333 are conserved among P RNAs as purines (Fig. 7B) although U occurs at G332 in P RNA from *Thermotoga* species. The nucleotide sequence AGA in J18/2 is universally conserved as is the sequence AU which flanks A333 and G332 on either side. These residues are involved in tertiary contacts within the folded P RNA structure and are distal to the bound substrate relative to A333 and G332. Therefore, we initially examined the effects of A248U, G332U, and A333U mutations on 5′Cy3-MD1 cleavage by quantifying product formation using PAGE.

The results showed that an A248U mutation results in a dramatic decrease in MD1 product formation, consistent with previous studies (Fig. 7C).^20,66^ The slow reaction rate is in line with the expectation based on previous results of a 300-fold increase in *K*_d_ that would result in a predicted value of *ca.* 40 μM for MD1.^20^ However, recent studies of A248U under conditions optimal for catalysis by P RNA alone (0.8 M ammonium acetate, 100 mM Mg^2+^) increase sensitivity to T1 cleavage in P18 consistent with an indirect effect on function due to local misfolding.^66^ Surprisingly, we observed that an A333U mutation has minor effects on *K*′ and *k*_c_, consistent with the residue's structural importance but not indicative of fundamental substrate interactions nor an essential role for a purine at this position. Similarly, G332U single turnover kinetics are essentially indistinguishable from native *E. coli* RNase P. These results are consistent with a role for A248 in positioning the reactive phosphate in the active site. The results suggest only minor roles for specific functional groups of G332 and A333 in substrate binding and are more generally consistent with a role for A333 in stacking.

### Mutations in P4 uncouple binding and catalysis

Mutations in P4 that alter the position of the bulged nucleotide U69 in P4 dramatically reduce RNase P catalysis that is linked to binding of active site divalent ions.^27–31^ Despite important steps forward in pinpointing key functional groups in P4 and the C-domain involved in Mg^2+^ binding, previous studies focused primarily on the contribution to the chemical step and not effects on binding. Moreover, most data are derived from the reactions of the P RNA subunit alone under high monovalent or divalent ion conditions or using engineered non-native constructs.

Therefore, to further resolve the contribution of U69 and P4 to binding kinetics and catalysis we investigated previously characterized P4 mutations (U69A and C70U) on MD1 cleavage by the *E. coli* RNase P holoenzyme using PAGE and stopped flow assays. Previous studies with P RNA alone under high salt conditions showed that a U69A mutation does not alter the pattern of base-pairing in P4 and results in only a fivefold reduction in *k*_c_.^30,68^ This result suggests the a key role of U69 in maintaining the bulged helical structure of P4. However, a 4-thiouridine modification of U51, the homologous residue in *B. subtilis* P RNA, had a much larger effect on catalysis^29^ underscoring the need for further information on its role. A C70U mutation was identified by *in vitro* selection for *E. coli* P RNA with faster reactivity in Ca^2+^*versus* Mg^2+^ from a pool of ribozymes randomized in P4.^69^ However, these studies used a fused self-cleaving RNase P-tRNA construct in high monovalent ion conditions (2 M), and did not include quantitative studies on ptRNA processing or holoenzyme reactions. Thus, a more detailed investigation of these mutants can reveal important new features of RNase P active site divalent ion specificity.

**Fig. 8:**
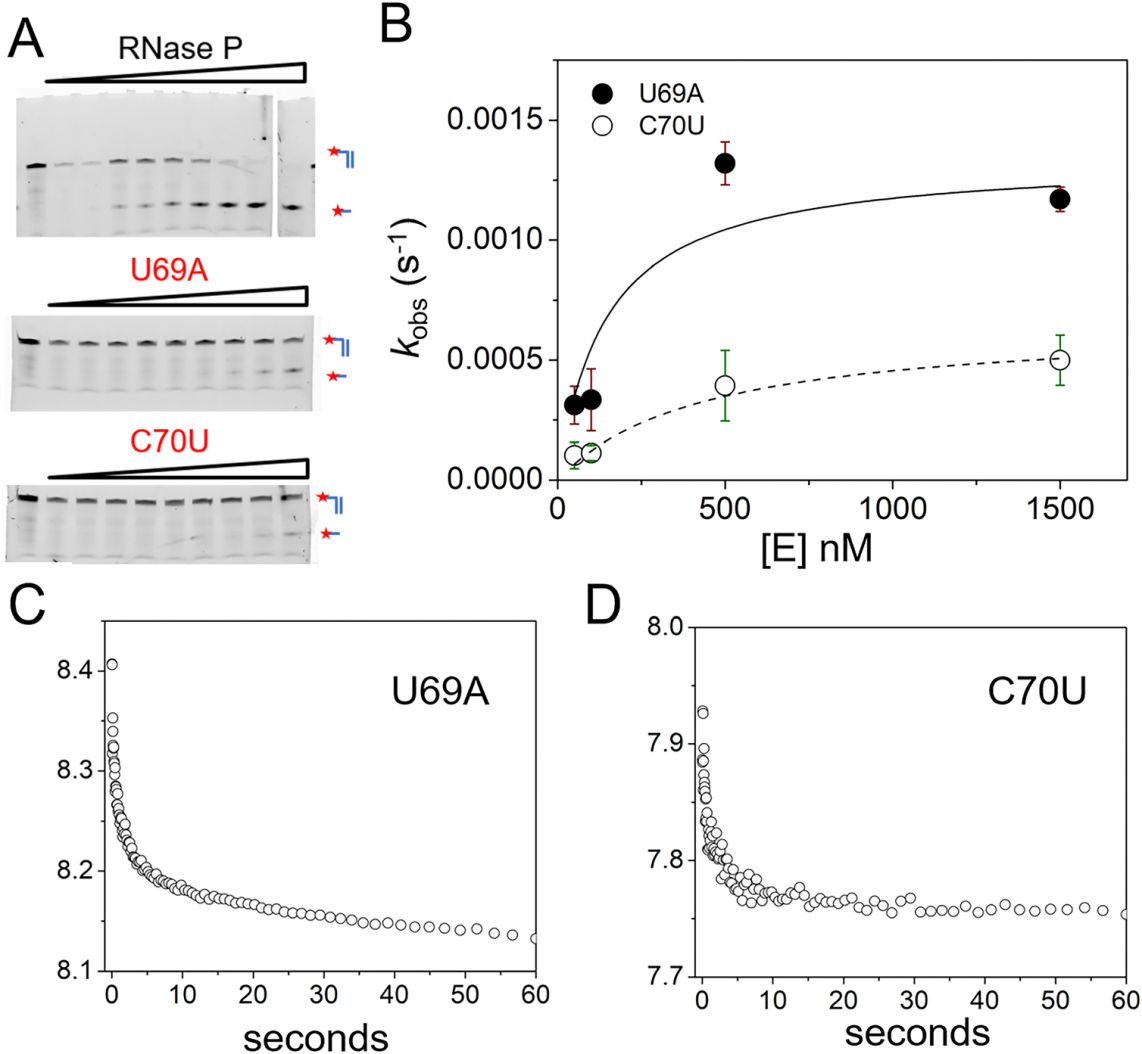
Effects of U69A and C70U mutation on *E. coli* RNase P binding and cleavage kinetics. (A) Analysis of the cleavage products of 5′Cy3-MD1 by PAGE for reactions with RNase P mutants U69A and C70U. (B) Plot of *k*_obs_ for the single turnover reactions of 5′-Cy3-MD1 as a function of enzyme concentration: U69A (filled circles), C70U (open circles). The data are fit to eqn (2) for Scheme 1 as described in Methods (Table 1). (C) and (D) Stopped flow fluorescence experiment. 2Ap(-2)-MD1 and mutant *E. coli* RNase P U69A (C) and C70U (D) were mixed to final concentrations of 100 nM and 1 μM, respectively.

To quantify the effects of U69A and C70U mutation on binding affinity and catalysis we used single turnover reactions to estimate *k*_c_ and *K*′. We also used stopped-flow fluorescence for analysis of the kinetics of 5′Ap(-2)-MD1 to measure association and cleavage kinetics. PAGE analyses of reaction products was consistent with cleavage at the correct site although low substrate conversion precludes detection of minor cleavage products (Fig. 8A). In single turnover reactions the *k*_obs_ was saturated at relatively low enzyme concentrations for both mutants consistent with minimal effects on substrate binding (Fig. 8B). The effects of both mutations on catalysis were significant under these conditions with U69A resulting in a *ca.* 10-fold decrease in *k*_c_ and C70U a *ca.* 250-fold reduction. The data are consistent with a *K*′ of less than 250 nM which would be in the range of the wild type enzyme. However, the very slow rate constants at low enzyme concentrations (<0.0001 s^−1^) were difficult to measure with precision limiting our ability to quantify *K*′ for these mutants. Therefore, we analyzed the binding and cleavage of C70U and U69A mutant RNase P enzymes by stopped flow fluorescence using the same 5′Ap(-2)-MD1 substrate (Fig. 8C and D). Both U69A and C70U mutants showed the initial rapid decrease in fluorescence associated with substrate binding. These data fit a single exponential function with observed rate constants of 0.32 s^−1^ and 0.35 s^−1^ for U69A and C70U, respectively, that are comparable to the value of 0.16 s^−1^ for *k*_obs1_ for native RNase P measured under the same conditions (Fig. 3B). Thus, the large defects in the substrate cleavage step engendered by U69A and C70U mutation are independent from the change in 2Ap fluorescence at N_−2_ that is induced by RNase P association.

### Evidence that slow catalysis in Ca^2+^ is due to altered active site structure

**Fig. 9:**
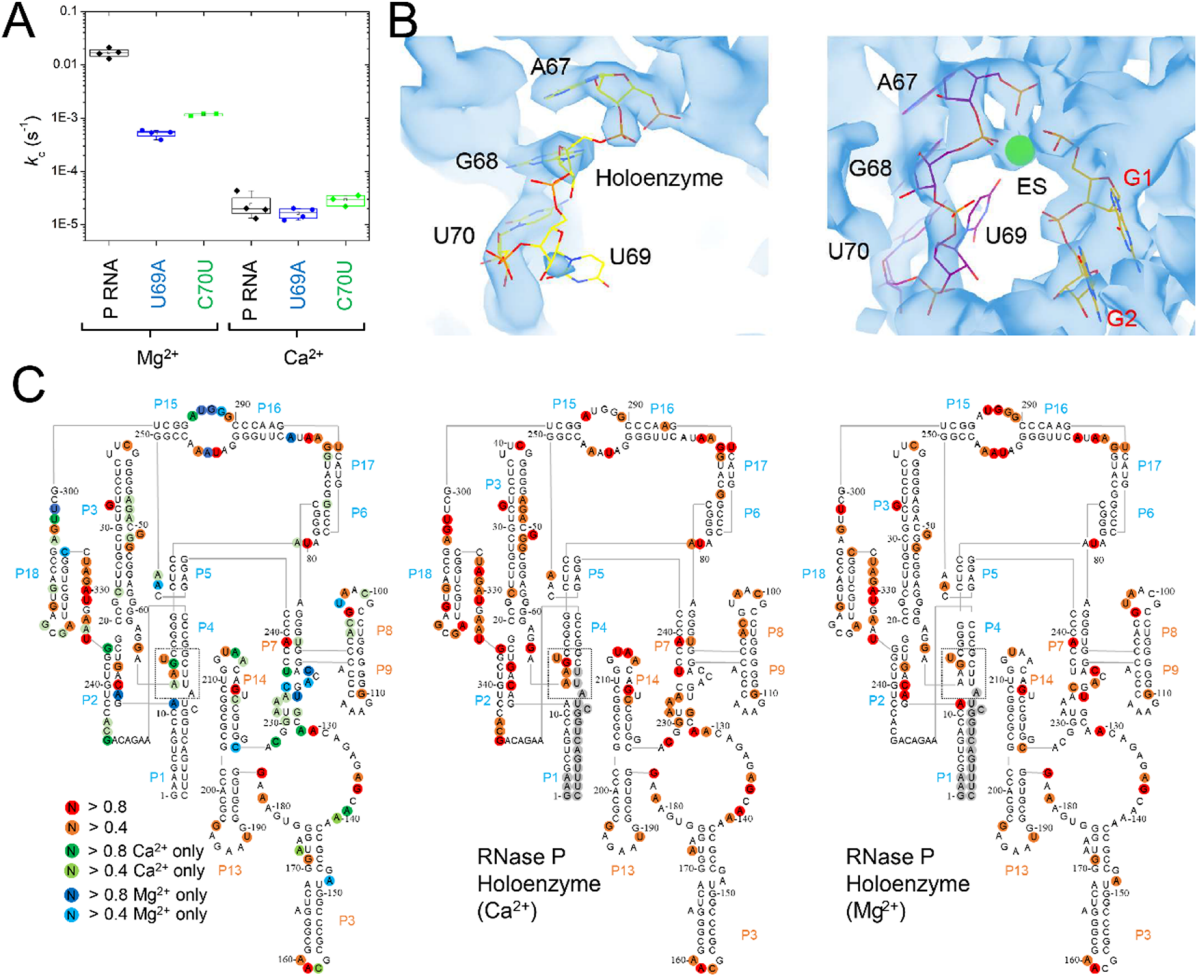
Differential effects of C70U and U69A mutations on P RNA structure and catalysis in the presence of Mg^2+^*versus* Ca^2+^. (A) Single turnover kinetics (*k*_2_) for native (WT), C70U, and U69A mutant RNase P enzymes in the presence of Mg^2+^ or Ca^2+^. The data are shown on a log scale. (B) CryoEM density map and structure model of the active site of *E. coli* RNase P enzyme–substrate complex (PDB: 7UO5). The positions of G1, A67, G68, and U69 are indicated, the green sphere is an active site metal. (C) SHAPE reactivity analysis of the modification patterns observed for *E. coli* P RNA in the presence of Mg^2+^*versus* Ca^2+^. The color of the circles for individual nucleotides reflects the normalized modification score with dark colors indicating higher reactivity. Red/orange circles indicate sites of modification observed in both Mg^2+^ and Ca^2+^. Green/light green circles show the sites of modification observed only in Ca^2+^, while the blue/light blue circles indicate the positions detected only in P RNA folded in Mg^2+^.

The C70U mutation was isolated by Pace and colleagues from a SELEX experiment and a self-cleaving P RNA–ptRNA conjugate designed to select for P RNA mutants with faster cleavage rates in Ca^2+^.^69^ C70U mutation resulted in a similar *k*_obs_ for reactions in Ca^2+^*versus* Mg^2+^, but also caused a decrease in activity independent of divalent ion type. These experiments were performed with a non-native construct, with the RNA alone, under high monovalent ion concentration (1 M). To better understand the functional effects of the C70U mutation on metal ion specificity we measured the *k*_c_ for the C70U and U69A mutants in the context of the *E. coli* RNase P holoenzyme in Ca^2+^ under saturating enzyme concentrations (Fig. 9A). Surprisingly, the effect of both mutations on the observed *k*_c_ for MD1 reactions in Ca^2+^ was relatively small compared to Mg^2+^.

The lack of effect of U69 mutations on *k*_c_ in Ca^2+^ could be due to a change in the rate-limiting step, or a difference in the role of U69 in active site interactions involving Ca^2+^*versus* Mg^2+^. Previous biochemical data and cryoEM studies showed that the structure of *E. coli* RNase P holoenzyme is essentially identical in both Mg^2+^ and Ca^2+^, although one or more changes in critical structural elements or cumulative small effects due to differences throughout the P RNA are possible. Among these differences we noted that U69 has weak cryoEM map features in both the free *E. coli* RNase P holoenzyme and the ES complex when Ca^2+^ is used as the divalent ion (Fig. 9B). This observation suggests that the insensitivity of U69 to mutation in Ca^2+^ could be due to its increased flexibility in Ca^2+^ and failure to productively engage in divalent ion interactions.

To further investigate the flexibility of U69 and differences in P RNA structure in Mg^2+^ and Ca^2+^ we performed SHAPE mapping of *E. coli* P RNA (Fig. 9C).^70,71^ The overall pattern of modification in both divalent ions occurred in loops, bulges, and single-stranded regions that are consistent with the folded structure of P RNA. There were 46 modifications sites that were common in both Mg^2+^ and Ca^2+^ while 38 additional modifications were detected in Ca^2+^ compared to 15 nucleotides where SHAPE reactivity was greater in Mg^2+^. Both common and Mg^2+^- or Ca^2+^-specific modifications sites were detected throughout *E. coli* P RNA. In some regions the results are consistent with overall higher reactivity in Ca^2+^ with additional modifications detected at nucleotides adjacent to positions where SHAPE reactivity occurs in both ions (*e.g.* 47–50 in P3; 93–98 in L8; 214–215 in L14; and 314–316 in L18). However, there were three regions (J5/15, P15–P16, and P4) in the C-domain of P RNA where the pattern of reactivity changed within structures that form the active site.

The most extensive difference in modification pattern occurred in the P15–16 internal bulge that binds the 3′RCC sequence of ptRNA where reactivity observed in Mg^2+^ was suppressed by Ca^2+^ (292–294) except of A295 which showed an increase in modification in the presence of Ca^2+^. P15–16 has been shown to bind divalent ions and contribute to interactions that are important for RNase P catalysis. Based on available structures of bacterial RNase P bound to ptRNA and tRNA, however, it does not appear as though P15–P16 directly contacts ions that also interact with the reactive phosphoryl group. A shift in the reactivity in J5/15 is observed as well from A248 in Mg^2+^ to A249 in Ca^2+^. Also, in the presence of Ca^2+^, there is increased reactivity in P4 at G68 and A66 and suppression of reactivity observed with Mg^2+^ at A11 in the P1–P4 helical junction proximal to U69. Thus, both cryoEM data and SHAPE mapping are consistent with greater flexibility of U69 in the presence of Ca^2+^. This greater flexibility correlates with the 300-fold slower *k*_c_ observed in Ca^2+^ relative to Mg^2+^ which is likely due, at least partially, to a reduced ability of Ca^2+^ to engage U69 in binding interactions. This result suggests a rationale for the minimal effects of U69A and C70U mutations on *k*_c_ in Ca^2+^*versus* Mg^2+^ in which the effects of mutation seen at U69 in the presence of Mg^2+^ is suppressed in the presence of Ca^2+^ because the latter cannot cooperate with U69 in promoting catalysis.

## Discussion

Here we report the detailed reaction kinetics of a minimal duplex substrate for bacterial RNase P with dual internal and terminal fluorescent probes and illustrate its use for gel-based assays as well as equilibrium and stopped flow fluorescence spectroscopy. As illustrated previously for similar minimal substrates for bacterial RNase P,^49^ MD1 is further adaptable to fluorescence based plate reader assays for high throughput screening and follow on mechanism of action studies. As an initial application we employed MD1 to quantify the effects of mutations at the key P RNA active site residues, and revealed that, contrary to previous models, G332 and A333 are unlikely to participate in sequence-specific interactions with the MD1 5′ leader.^10^ We further demonstrated that mutations at U69 in P4, a key active site metal ion contact, uncouples substrate binding and catalysis. Surprisingly, the effects of U69 mutation on catalysis were minimized when Ca^2+^ replaced Mg^2+^ in the reaction, which correlates with greater flexibility of U69 in Ca^2+^ observed by cryoEM density analysis and SHAPE. Taken together, these new data suggest that stabilizing interactions involving U69, that occur with Mg^2+^ that are susceptible to mutation, are missing in Ca^2+^. This result runs counter to previous mechanistic interpretation of slower RNase P catalysis in Ca^2+^ relative to Mg^2+^ in terms of chemical differences in nucleophilic activation.^62,72^ The results further demonstrate a facile analytical tool for dissecting active site interactions and the involvement of key enzyme and substrate functional groups using chemical and isotopic substitutions for mechanistic studies.

Minimal versions of tRNA have been widely used to investigate the structure and function of tRNA modifying enzymes and tRNA binding proteins. In 1989 Franklyn and Shimmel showed that a “minihelix” derivative of tRNA composed of the stacked amino-acid acceptor-TΨC stems was a substrate for alanine tRNA synthetase.^73^ Other minimal minihelix substrates consisting of the acceptor and anticodon stems as well as miniduplexes like MD1 have also been developed as tools for analysis of aminoacyl tRNA synthetases structure and function *e.g.*,^74,75^ as well as EF-Tu.^76,77^ They have continued to serve as useful tools for these and other enzymes and tRNA binding proteins.^78,79^

Similar minihelix or miniduplex substrates have been successfully used to investigate bacterial RNase P and provided significant insights into interactions at the cleavage site, roles and interactions of divalent ions, and principles for engineering P RNA for therapeutic applications. Using SELEX to discover novel substrates Pan and colleagues used a circular RNA pool to discover short stem loop and duplex substrates for P RNA ribozyme reactions, thus demonstrating inherent specificity for such alternative substrates.^80^ Small bipartite model substrates were shown to be substrates for *E. coli* and *T. thermophilus* RNase P enzymes, however, there are significant defects in processing due to sequence length and structure.^44^ Even smaller 3bp stems have been used to probe the contribution of TPsiC stem loop interactions.^34,48^ Acceptor stem duplexes containing only a three-nucleotide 5′ leader are efficient minimal substrates for *E. coli* and *B. subtilis* RNase Ps enabling biophysical studies of nucleotide conformations and metal ion interactions at the cleavage site.^53,81^ With respect to probing active site interactions a model hairpin-loop substrate pATSer has been extensively used by the Kirsebom laboratory, initially to investigate the roles of metal ions in catalysis and identify nucleotides and functional groups involved in 5′ and 3′ end recognition.^11,13^ These studies took full advantage of the ability to site-specifically embed functional group modifications at the atomic level to probe the interactions at the substrate cleavage site.^38,39,45,48,59,82^ Using pATSer these researchers showed the importance of N_−1_ the N_1_–N_73_ base pair and importance of the T-loop sequence for PRORP.^83^

Duplex and minimal substrates have also been adopted to engineer RNase P specificity and for drug discovery. *E. coli* P RNA fused to additional 3′ guide sequences results in the potential to form an intermolecular duplex substrate that can be used to target RNAs for cleavage through hybridization with target sequences.^51,84^ These guide RNA containing P RNAs (M1GS) have been shown to provide useful tools to hydrolyze human and viral mRNAs in order to decrease gene expression and virus replication.^85^ For discovery of small molecule inhibitors Torres–Larios and colleagues used a small minihelix substrate incorporating an Alexa fluorophore and quencher on the 3′ and 5′ ends, respectively, for which cleavage results in a signal-on assay for high-throughput screening. A key advantage of such minimal substrates is avoidance of potential false positives due to small molecules that inhibit the reaction by binding to structural elements of tRNA instead of binding to the target enzyme RNase P, although the loss of interactions important for ptRNA binding when using minimal substrates could engender some risk of false negatives (*e.g.* loss of small molecules that bind to the S domain of RNase P RNA to inhibit interaction with the tRNA T loop).^49^ Thus, the information gained from detailed kinetics of minimal and alternative substrates can be useful in engineering the P RNA ribozyme activity for therapeutic applications and deeper understanding of effects of inhibitors on reaction kinetics. Our studies using MD1 build directly on the success of these studies by incorporating terminal and internal fluorescent probes allowing multiple modes of detection. This capability permits direct comparison of results across a range of methods including traditional kinetics assays using PAGE, rapid kinetics, and high throughput assays.

The analysis of a set of previously characterized active site mutants using the MD1 substrate are consistent with the well-established roles of A248 and U69 in bacterial RNase P enzyme function. Biochemical and structural data indicate direct interaction between nucleotides G332 and A333 in J18/2 and nucleotides proximal to the cleavage site.^10,16,35,86,87^ Yet, mutating these conserved purines to U did not provide evidence for significant contributions to 5′ leader recognition or catalysis. Based on miscleavage results observed using substrates containing a 2′-deoxy modification at N_−1_ we proposed that A248 forms a *cis* WC/WC base pair with N_−1_.^20^ However, studies aimed at testing N_−1_ and A248 interactions using extensive chemical modification of minimal substrates showed that sensitivity to modification was substrate dependent, but largely inconsistent with a *cis* WC/WC interaction.^66^ The cryoEM structure of *E. coli* RNase P bound to two alternative substrates with different nucleotides at N_−2_/N_−1_ revealed that for both dinucleotides the WC edge of N_−1_ in fact contacts the Hoogsteen edge of A248 while N_−2_ is sandwiched between N_−1_ and A333 such that a continuous three nucleotide helical stacking geometry forms in the order 5′- A333/N_−2_/N_−1_-3′. Thus, mutation of either G332 or A333 to U should not interfere with a role in stacking interactions that position the substrate in the active site. However, the results with MD1 alone do not rule out sequence specific interactions between G332 and A333 for some alternative substrates.

The central role of P4 and the universally conserved bulged U in binding catalytic metal ions is well established. However, key questions remain regarding the number and roles of divalent ions involved directly in transition state stabilization. The model for RNase P catalysis is based on a chemically feasible two metal ion model and kinetic assays as well as biophysical studies support the participation of two or more divalent ions in transition state stabilization. However, most previous studies of P4 metal ion interactions were performed using the P RNA ribozyme alone, typically at high monovalent and divalent ion concentrations. Although foundational these conditions could obscure or enhance interactions important in holoenzyme reactions. Also, since these studies focused on the role in catalysis, they used single turnover reaction conditions to minimize the contributions of substrate binding. Taking advantage of the ability to measure rapid kinetics we demonstrate that mutations at U69 in P4 resulting in large defects in catalysis nonetheless have minimal impact on the rate constant for substrate binding. This result suggests that Mg^2+^ interactions involving U69 in the transition state may not necessarily be populated in the ground state pre-catalytic complex (ES*). The current mechanistic model for RNase P binding involves a conformational change that forms the coordination site for an essential but weakly binding catalytic metal ion. The results here are consistent with even weaker binding of this ion or class of ions due to U69 mutation resulting in accumulation of ES* with minimal effects on the steps for its formation.

Interestingly, the effects of mutation, previously indicated to affect the ability of U69 to participate in catalytic metal ions interactions, diminish in Ca^2+^. As an alternative divalent ion Ca^2+^ has been used widely in the investigation of RNase P to slow the cleavage step without large effects on the structure of the ES* complex. This interpretation is supported by biochemical studies^62,88–92^ and comparison of the crystal structure of *T. maritima* RNase P solved in Mg^2+^ and cryoEM structure of *E. coli* RNase P ES* complex assembled in Ca^2+^.^15,16^ However, the mechanistic basis for slower cleavage in Ca^2+^ has not been established. Early studies which demonstrated the use of substituting Ca^2+^ to slow cleavage and distinguish binding and catalysis suggest a connection with divalent ion p*K*_a_. The higher p*K*_a_ for deprotonation of a water molecule in the hydration sphere of Ca^2+^ compared to Mg^2+^ (12.7 *versus* 11.4) provided a rationale for slower catalysis as the direct result of lower concentration of metal-hydroxide which acts as a nucleophile or general base to accept a proton from water.

However, the results presented here together with previous cryoEM and mutagenesis data provide an alternative explanation for slower catalysis in Ca^2+^ and why this effect is uncoupled from binding. CryoEM data and SHAPE analysis of *E. coli* P RNA are consistent with greater flexibility of U69 in the free holoenzyme and that persists in the ES* complex. This greater flexibility suggests that Ca^2+^ fails to engage in productive interactions with U69 as the cause of slower cleavage. As demonstrated by stopped flow analysis, both U69A and C70 showed native substrate binding kinetics in Mg^2+^ despite large effects on catalysis. Since the metal ion interactions involving U69 contribute primarily in the transition state, the inability of Ca^2+^ to engage U69 productively does not affect binding. Similarly, mutations altering U69 function in Mg^2+^ have little effect on catalysis in Ca^2+^ because it makes a different or restricted set of interactions in which U69 participates less. That is, the stabilizing interactions involving U69 that occur with Mg^2+^ that are susceptible to mutation are missing in Ca^2+^.

## Methods

### Preparation of RNAs and holoenzyme assembly

RNA oligonucleotides (Table 1) were obtained commercially (Horizon Dharmacon) and deprotected according to vendor protocol. The 3′MD1 RNA oligo was used for all MD1 substrates which differed according to the desired 5′ fluorescent probe modification (5′Ap(-2)-MD1; 5′Cy3-MD1, 5′Cy3Ap(-2)-MD1, or 5′Cy3Ap(-2)BHQ-MD1). To prepare duplex MD1 substrates the 3′MD1 RNA was combined with the appropriate complementary RNA at a ratio of 1 : 1–1 : 2 in reaction buffer (MES pH 6 or Tris pH 8; 100 mM NaCl, 0.15% Triton *x*-100) and incubated at 95 °C for 3 minutes then transferred to 37 °C for 10 minutes. An aliquot of concentrated MgCl_2_ or CaCl_2_ solution was added to achieve the desired concentration, and the incubation continued for an additional 10 min at 37 °C. The *E. coli* RNase P RNA subunit was synthesized by *in vitro* transcription using T7 polymerase from a plasmid DNA template, and purified by one of two methods-denaturing PAGE or native size exclusion chromatography (SEC). The denaturing urea-PAGE method used 6% polyacrylamide gels in TBE buffer, visualization by UV shadowing, and passive elution of the excised RNA band into 40 mM Tris pH 7.5, 10 mM EDTA, 1% SDS. Eluted RNA was recovered by ethanol precipitation after phenol/chloroform extraction.^93^ For the SEC method, P RNA was purified after *in vitro* transcription using an AKTA PURE instrument and Hiload 16/600 Superdex 200 pg column with isocratic elution TE buffer (10 mM Tris pH 7.5, 1 mM EDTA). The eluted RNA was stored at −20 °C.

The *E. coli* C5 protein was affinity purified using the NEB IMPACT system. The C5 protein was overexpressed as a chitin-binding domain fusion with subsequent removal of the tag by intein cleavage followed by dialysis and concentration as previously described.^57^ P RNA purified from *in vitro* transcription was incubated at 95 °C for 3 minutes in reaction buffer (MES pH 6 or Tris pH 8; 100 mM NaCl, 0.15% Triton *x*-100), followed by incubated at 37 °C for 10 minutes. MgCl_2_ or CaCl_2_ was added, and the incubation continued at 37 °C for another 10 minutes. The RNase P holoenzyme was assembled by adding C5 at a ratio of 1 : 1.5 and further reaction for 10 minutes at 37 °C.^57,60^

### Single turnover kinetics performed using PAGE electrophoresis

Single turnover reactions were performed in 50 mM MES pH 6, 100 mM NaCl, 0.015% Triton-X reduced, 17.5 mM MgCl_2_ at 37 °C and used RNase P holoenzyme concentrations ranging from 25 nM–1.5 μM. For reactions containing 250 nM or more RNase P the final substrate concentration was 25 nM. For RNase P concentrations of 100 nM or less the final substrate concentration was lowered to 8–10 nM. Reactions were initiated by mixing equal volumes of RNase P and ptRNA stock solutions at twice the desired concentration. Aliquots of the reaction mixture were removed and quenched by combining with an equal volume of 90% formamide, 100 mM EDTA. The precursor and product were then separated by 15% denaturing PAGE and detected using an Amersham Typhoon RGB phosphorimager (GE). The substrate and product bands were then quantified using ImageQuant TL 8.2 (GE) to calculate the fraction of total substrate converted to product. The observed rate constant *k*_obs_ was determined by plotting the fraction of product formed (*y* = [P]/([P] + [S])) *vs.* time (*x*) and fitting the data to a single exponential function (eqn (1)) in Origin 8.5. The dependence of *k*_obs_ on [E] was analyzed assuming a rapid equilibrium mechanism (*k*_−1_ ≫ *k*_2_), using eqn (2), where *K*′ is the dissociation constant for MD1 binding to *E. coli* RNase P, and *k*_c_ is the first-order rate constant for conversion of ES to EP according to Scheme 1.

1 *y* = *a* + *b*e^−k_obs_*x*^

2
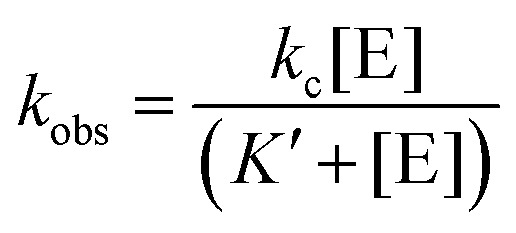


### Equilibrium and stopped-flow fluorescence spectroscopy

Steady-state spectra of MD1 assembled using the 5′Cy3Ap(-2)-MD1 RNA oligo were measured for the following samples. Substrate: Prepared as described above at a concentration of 100 nM of MD1. ES Complex: 100 nM MD1and 1 μM RNase P in reaction buffer containing 17.5 mM CaCl_2_. Enzyme plus Product: 100 nM MD1and 1 μM RNase P in reaction buffer containing 17.5 mM MgCl_2_. The Enzyme plus Product sample was incubated at 37 °C for 1 hour to achieve complete conversion of ptRNA. Samples were maintained at 37 °C for the duration of the experiment. Spectra were acquired using a thermostatted Carey Eclipse fluorescence spectrometer. Scans were taken in a 5 mm × 5 mm cuvette at 37 °C from 320–450 nm with an excitation wavelength of 305 nm, emission slits at 5 nm, and a scan rate of 120 nm min^−1^. Three spectra were averaged, and an averaged spectrum of a background control was subtracted to yield the final spectra.

The time dependence of changes in the fluorescence of 2Ap and Cy3 labels in MD1 were measured by stopped-flow fluorescence spectroscopy using an Applied Photophysics SX20 instrument equipped with LED light sources (300 nm for 2Ap, and 535 nm for Cy3) in a single mixing (two syringe) mode. Power supply outputs were varied between 2 and 12 mA depending on the duration of the kinetics to optimize sensitivity and minimize photobleaching. Photobleaching was evaluated by comparing rate constants obtained at different LED outputs. Emission was measured using a high pass filter (320 nm for 2Ap and 570 nM for Cy3). Temperature was maintained at 37 °C using a circulating water bath connected to the sample handling unit. Equal volumes of RNase P and MD1 solutions, each at 2× the respective final concentration, were mixed to yield a total drive volume of 140 μL. Stopped flow kinetic were initially analyzed using a double exponential function to estimate the rate constants for the first and second reaction phases.

3 *y* = *a* + *b*1e^−*k*_obs1_*x*^ + *b*2e^−k_obs2_*x*^

Global data fitting and analysis were performed using KinTek Explorer v11 (KinTek).^94^ Fluorescence anisotropy measurements were performed with the instrument's T-format excitation and emission polarizer accessory. Samples were excited using the 535 nm LED and the vertical and horizontal polarized light emission was monitored at 90° using 570 nm long-pass filters on both detectors. To correct for gain differences in the two detection channels a *G*-factor was determined prior to running the experiments using the SX software. The observed rate constant reflecting binding was determined using the single exponential function (eqn (1)) in Origin 8.5.

### Fluorescence microplate reader assay of multiple turnover kinetics

The fluorescence signal of the 5′Cy3Ap(-2)BHQ-MD1 RNA substrate is quenched due to the presence of a 3′ modification with BHQ2. To measure this signal for multiple turnover kinetics we used a BMG Clariostar microplate reader and mixed separate solutions at twice the intended final concentration using the instrument's injector. Final substrate concentrations were 50 nM–5 μM 5′Cy3Ap(-2)BHQ-MD1 and 10 nM *E. coli* RNase P. First, 30 μL of 2*x* substrate in Tris pH 8; 100 mM NaCl, 0.15% Triton *x*-100 was pipetted into the wells of a 90 well black low-bind microplate (Corning 3684). A range of concentrations were used to survey substrate limiting and saturating conditions (50 nm–5 μM). To start the reactions 30 μL of 2*x* enzyme solution in the same buffer was injected into each well that contained substrate. After 5 seconds of shaking to mix the solutions, the Cy3 fluorescence intensity was measured for each well with a cycle time of 30s. The initial and final fluorescence intensities were determined by fitting the data to eqn (1). Using these values the amplitude was normalized and the first 10% of the data was fit to a linear function and the slope determined by fitting was used to calculate *v*_obs_ in units of nM s^−1^ for each substrate concentration.

### *In vitro* SHAPE analysis

DNA oligonucleotides for primer extension were 5′ end labeled with γ-^32^P-ATP and polynucleotide kinase followed by gel purification by denaturing urea-PAGE on 20% polyacrylamide gels. SHAPE reactions contained 100 nM RNase P and 500 nM ptRNA was added to probe the structure of the ES complex. Binding reactions in standard reaction buffer containing Ca^2+^ or Mg^2+^ as indicated in the text were conducted. 5 μL of *N*-methylisatoic anhydride (NMIA) stock was added to 45 μL RNase P in Mg^2+^, or Ca^2+^, of the RNase P–ptRNA complex in Ca^2+^. The modified RNA was recovered by ethanol precipitation, and resuspended in 10 μL 10 mM Tris pH 7.5 and 1 mM EDTA for subsequent primer extension mapping.^70^ The samples were resolved using 8–10% denaturing PAGE gels, then scanned by phosphorimager and individual band intensities were quantified using the SAFA (Semi-Automated Footprinting Analysis) program.^95^ The absolute NMIA reactivity for each sample was measured at each position by subtracting the no NMIA control intensities band intensities of the NMIA reacted samples intensities. The control and NMIA reacted intensities were normalized assuming low intensity reflects unreactive positions, and common thresholds for high and low reactivity for the two data sets were established as described.^71^

## Author contributions

MEH – supervision, conceptualization, investigation, data curation, writing & review. TH – conceptualization, investigation, data curation, visualization, writing & review. AC – conceptualization, investigation, data curation, visualization, writing & review. JZ – investigation, data curation, writing & review.

## Conflicts of interest

The authors have no conflicts of interest to declare.

## References

[cit1] Hartmann R. K., Heinrich J., Schlegl J., Schuster H. (1995). Proc. Natl. Acad. Sci. U. S. A..

[cit2] Altman S., Wesolowski D., Guerrier-Takada C., Li Y. (2005). Proc. Natl. Acad. Sci. U. S. A..

[cit3] Li Y., Cole K., Altman S. (2003). RNA.

[cit4] Li Y., Altman S. (2003). Proc. Natl. Acad. Sci. U. S. A..

[cit5] Mohanty B. K., Kushner S. R. (2018). Microbiol Spectr.

[cit6] Mohanty B. K., Agrawal A., Kushner S. R. (2020). Nucleic Acids Res..

[cit7] Guerrier-Takada C., Gardiner K., Marsh T., Pace N., Altman S. (1983). Cell.

[cit8] Lai L. B., Zahurancik W. J., Gopalan V. (2021). Trends Biochem. Sci..

[cit9] Lai L. B., Vioque A., Kirsebom L. A., Gopalan V. (2010). FEBS Lett..

[cit10] Klemm B. P., Wu N., Chen Y., Liu X., Kaitany K. J., Howard M. J., Fierke C. A. (2016). Biomolecules.

[cit11] Kirsebom L. A., Trobro S. (2009). IUBMB Life.

[cit12] Chamberlain A. R., Huynh L., Huang W., Taylor D. J., Harris M. E. (2023). J. Biol. Chem..

[cit13] Kirsebom L. A. (2007). Biochimie.

[cit14] Klemm B. P., Karasik A., Kaitany K. J., Shanmuganathan A., Henley M. J., Thelen A. Z., Dewar A. J. L., Jackson N. D., Koutmos M., Fierke C. A. (2017). RNA.

[cit15] Reiter N. J., Osterman A., Torres-Larios A., Swinger K. K., Pan T., Mondragón A. (2010). Nature.

[cit16] Zhu J., Huang W., Zhao J., Huynh L., Taylor D. J., Harris M. E. (2022). Nat. Commun..

[cit17] Burgin A. B., Pace N. R. (1990). EMBO J..

[cit18] Siew D., Zahler N. H., Cassano A. G., Strobel S. A., Harris M. E. (1999). Biochemistry.

[cit19] Zahler N. H., Sun L., Christian E. L., Harris M. E. (2005). J. Mol. Biol..

[cit20] Zahler N. H., Christian E. L., Harris M. E. (2003). RNA.

[cit21] Brännvall M., Fredrik Pettersson B. M., Kirsebom L. A. (2002). Biochimie.

[cit22] Kirsebom L. A., Svärd S. G. (1994). EMBO J..

[cit23] Busch S., Kirsebom L. A., Notbohm H., Hartmann R. K. (2000). J. Mol. Biol..

[cit24] Brännvall M., Pettersson B. M., Kirsebom L. A. (2003). J. Mol. Biol..

[cit25] Oh B. K., Pace N. R. (1994). Nucleic Acids Res..

[cit26] Hardt W. D., Schlegl J., Erdmann V. A., Hartmann R. K. (1995). J. Mol. Biol..

[cit27] Christian E. L., Kaye N. M., Harris M. E. (2002). EMBO J..

[cit28] Christian E. L., Kaye N. M., Harris M. E. (2000). RNA.

[cit29] Liu X., Chen Y., Fierke C. A. (2017). J. Am. Chem. Soc..

[cit30] Kaye N. M., Zahler N. H., Christian E. L., Harris M. E. (2002). J. Mol. Biol..

[cit31] Crary S. M., Kurz J. C., Fierke C. A. (2002). RNA.

[cit32] Hsieh J., Fierke C. A. (2009). RNA.

[cit33] Hsieh J., Koutmou K. S., Rueda D., Koutmos M., Walter N. G., Fierke C. A. (2010). J. Mol. Biol..

[cit34] Brännvall M., Kikovska E., Wu S., Kirsebom L. A. (2007). J. Mol. Biol..

[cit35] LaGrandeur T. E., Hüttenhofer A., Noller H. F., Pace N. R. (1994). EMBO J..

[cit36] Rueda D., Hsieh J., Day-Storms J. J., Fierke C. A., Walter N. G. (2005). Biochemistry.

[cit37] McClain W. H., Guerrier-Takada C., Altman S. (1987). Science.

[cit38] Brännvall M., Kirsebom L. A. (2001). Proc. Natl. Acad. Sci. U. S. A..

[cit39] Brännvall M., Kirsebom L. A. (1999). J. Mol. Biol..

[cit40] Brännvall M., Mattsson J. G., Svärd S. G., Kirsebom L. A. (1998). J. Mol. Biol..

[cit41] Wu S., Chen Y., Lindell M., Mao G., Kirsebom L. A. (2011). J. Mol. Biol..

[cit42] Wu S., Kikovska E., Lindell M., Kirsebom L. A. (2012). J. Mol. Biol..

[cit43] Zuleeg T., Hansen A., Pfeiffer T., Schubel H., Kreutzer R., Hartmann R. K., Limmer S. (2001). Biochemistry.

[cit44] Schlegl J., Fürste J. P., Bald R., Erdmann V. A., Hartmann R. K. (1992). Nucleic Acids Res..

[cit45] Brännvall M., Kirsebom L. A. (2005). J. Mol. Biol..

[cit46] Brännvall M., Kikovska E., Kirsebom L. A. (2004). Nucleic Acids Res..

[cit47] Kikovska E., Brännvall M., Kirsebom L. A. (2006). J. Mol. Biol..

[cit48] Wu S., Chen Y., Mao G., Trobro S., Kwiatkowski M., Kirsebom L. A. (2014). Nucleic Acids Res..

[cit49] Madrigal-Carrillo E. A., Diaz-Tufinio C. A., Santamaria-Suarez H. A., Arciniega M., Torres-Larios A. (2019). Nucleic Acids Res..

[cit50] Mao G., Srivastava A. S., Wu S., Kosek D., Lindell M., Kirsebom L. A. (2018). PLoS One.

[cit51] Liu F., Altman S. (1996). Nucleic Acids Res..

[cit52] Hansen A., Pfeiffer T., Zuleeg T., Limmer S., Ciesiolka J., Feltens R., Hartmann R. K. (2001). Mol. Microbiol..

[cit53] Zuleeg T., Hartmann R. K., Kreutzer R., Limmer S. (2001). J. Mol. Biol..

[cit54] Svärd S. G., Kirsebom L. A. (1992). J. Mol. Biol..

[cit55] Beebe J. A., Kurz J. C., Fierke C. A. (1996). Biochemistry.

[cit56] Rau M. J., Hall K. B. (2015). Methods Enzymol..

[cit57] Guo X., Campbell F. E., Sun L., Christian E. L., Anderson V. E., Harris M. E. (2006). J. Mol. Biol..

[cit58] Smith D., Pace N. R. (1993). Biochemistry.

[cit59] Kikovska E., Brännvall M., Kufel J., Kirsebom L. A. (2005). Nucleic Acids Res..

[cit60] Yandek L. E., Lin H. C., Harris M. E. (2013). J. Biol. Chem..

[cit61] Niland C. N., Zhao J., Lin H. C., Anderson D. R., Jankowsky E., Harris M. E. (2016). ACS Chem. Biol..

[cit62] Smith D., Burgin A. B., Haas E. S., Pace N. R. (1992). J. Biol. Chem..

[cit63] Jones A. C., Neely R. K. (2015). Q. Rev. Biophys..

[cit64] Cleland W. W. (2005). Arch. Biochem. Biophys..

[cit65] Kurz J. C., Fierke C. A. (2002). Biochemistry.

[cit66] Mao G., Srivastava A. S., Wu S., Kosek D., Kirsebom L. A. (2023). Sci. Rep..

[cit67] Baer M. F., Reilly R. M., McCorkle G. M., Hai T. Y., Altman S., RajBhandary U. L. (1988). J. Biol. Chem..

[cit68] Tanaka T., Ando T., Haga S., Kikuchi Y. (2004). Biosci., Biotechnol., Biochem..

[cit69] Frank D. N., Pace N. R. (1997). Proc. Natl. Acad. Sci. U. S. A..

[cit70] Garfio C. M., Gupta M., Spitale R. C. (2023). Curr. Protoc..

[cit71] Smola M. J., Rice G. M., Busan S., Siegfried N. A., Weeks K. M. (2015). Nat. Protoc..

[cit72] Cassano A. G., Anderson V. E., Harris M. E. (2004). Biochemistry.

[cit73] Francklyn C., Schimmel P. (1989). Nature.

[cit74] Frugier M., Florentz C., Giegé R. (1992). Proc. Natl. Acad. Sci. U. S. A..

[cit75] Musier-Forsyth K., Schimmel P. (1994). Biochemistry.

[cit76] Nazarenko I. A., Uhlenbeck O. C. (1995). Biochemistry.

[cit77] Rudinger J., Blechschmidt B., Ribeiro S., Sprinzl M. (1994). Biochemistry.

[cit78] Ramaswamy K., Wei K., Suga H. (2002). Nucleic Acids Res..

[cit79] Schimmel P., Ribas de Pouplana L. (1995). Cell.

[cit80] Pan T. (1995). Biochemistry.

[cit81] Zuleeg T., Hansen A., Pfeiffer T., Schübel H., Kreutzer R., Hartmann R. K., Limmer S. (2001). Biochemistry.

[cit82] Kikovska E., Brännvall M., Kirsebom L. A. (2006). J. Mol. Biol..

[cit83] Mao G., Chen T. H., Srivastava A. S., Kosek D., Biswas P. K., Gopalan V., Kirsebom L. A. (2016). PLoS One.

[cit84] Förster A. C., Altman S. (1990). Science.

[cit85] Kim K., Liu F. (2007). Biochim. Biophys. Acta.

[cit86] Christian E. L., Harris M. E. (1999). Biochemistry.

[cit87] Christian E. L., McPheeters D. S., Harris M. E. (1998). Biochemistry.

[cit88] Hardt W. D., Schlegl J., Erdmann V. A., Hartmann R. K. (1993). Nucleic Acids Res..

[cit89] Kurz J. C., Niranjanakumari S., Fierke C. A. (1998). Biochemistry.

[cit90] Brännvall M., Mikkelsen N. E., Kirsebom L. A. (2001). Nucleic Acids Res..

[cit91] Koutmou K. S., Zahler N. H., Kurz J. C., Campbell F. E., Harris M. E., Fierke C. A. (2010). J. Mol. Biol..

[cit92] Christian E. L., Smith K. M., Perera N., Harris M. E. (2006). RNA.

[cit93] Nilsen T. W. (2013). Cold Spring Harb Protoc..

[cit94] Johnson K. A., Simpson Z. B., Blom T. (2009). Anal. Biochem..

[cit95] Simmons K., Martin J. S., Shcherbakova I., Laederach A. (2009). Methods Enzymol..

